# Cross-reactive antibodies targeting surface-exposed non-structural protein 1 (NS1) of dengue virus-infected cells recognize epitopes on the spaghetti loop of the β-ladder domain

**DOI:** 10.1371/journal.pone.0266136

**Published:** 2022-05-26

**Authors:** Romchat Kraivong, Somchoke Traewachiwiphak, Napon Nilchan, Nattaya Tangthawornchaikul, Nuntaya Pornmun, Ranyikar Poraha, Kanokwan Sriruksa, Wannee Limpitikul, Panisadee Avirutnan, Prida Malasit, Chunya Puttikhunt

**Affiliations:** 1 Molecular Biology of Dengue and Flaviviruses Research Team, National Center for Genetic Engineering and Biotechnology, National Science and Technology Development Agency, Pathum Thani, Thailand; 2 Medical Biotechnology Research Unit, National Center for Genetic Engineering and Biotechnology, Siriraj Hospital, Bangkok, Thailand; 3 Faculty of Medicine Siriraj Hospital, Siriraj Center of Research Excellence in Dengue and Emerging Pathogens, Mahidol University, Bangkok, Thailand; 4 Faculty of Medicine Siriraj Hospital, Division of Dengue Hemorrhagic Fever Research, Mahidol University, Bangkok, Thailand; 5 Pediatric Department, Khon Kaen Hospital, Ministry of Public Health, Khon Kaen, Thailand; 6 Pediatric Department, Songkhla Hospital, Ministry of Public Health, Songkhla, Thailand; National Research Council Canada, CANADA

## Abstract

Non-structural protein 1 (NS1) is a glycoprotein component of dengue virus (DENV) that is essential for viral replication, infection and immune evasion. Immunization with NS1 has been shown to elicit antibody-mediated immune responses which protect mice against DENV infections. Here, we obtained peripheral blood mononuclear cells from human subjects with secondary dengue infections, which were used to construct a dengue immune phage library displaying single-chain variable fragments. Phage selective for DENV NS1 were obtained by biopanning. Twenty-one monoclonal antibodies (mAbs) against DENV NS1 were generated from the selected phage and characterized in detail. We found most anti-NS1 mAbs used IGHV1 heavy chain antibody genes. The mAbs were classified into strongly and weakly-reactive groups based on their binding to NS1 expressed in dengue virus 2 (DENV2)-infected cells. Antibody binding experiments with recombinant NS1 proteins revealed that the mAbs recognize conformational epitopes on the β-ladder domain (amino acid residues 178–273) of DENV NS1. Epitope mapping studies on alanine-substituted NS1 proteins identified distinct but overlapping epitopes. Protruding amino acids distributed around the spaghetti loop are required for the binding of the strongly-reactive mAbs, whereas the recognition residues of the weakly-reactive mAbs are likely to be located in inaccessible sites facing toward the cell membrane. This information could guide the design of an NS1 epitope-based vaccine that targets cross-reactive conserved epitopes on cell surface-associated DENV NS1.

## Introduction

Dengue is a mosquito-borne infectious disease caused by dengue virus (DENV). DENV is a member of the *Flavivirus* genus, which includes other disease-causing viruses such as Japanese encephalitis virus and Zika virus (ZIKV). There are four dengue serotypes (DENV1–4) that share genetic and antigenic determinants. Primary infections of one serotype provide lifelong protection against subsequent homotypic infections, which are asymptomatic or present as mild dengue fever (DF). However, when the secondary infection is heterotypic, infected people can manifest severe dengue hemorrhagic fever (DHF), which can be fatal in certain cases [1]. One mechanism that can explain the enhancement of the disease severity in heterotypic infection is antibody-dependent enhancement (ADE), a phenomenon in which the antibodies generated from the previous infection do not protect, but instead enhance the ability of the virus to enter cells [2,3]. Although an approved dengue vaccine (Dengvaxia) is available, the vaccine is not recommended for DENV-naïve individuals as it could increase the risk of antibody dependent enhanced (ADE) in subsequent DENV infection [4,5]. To date, dengue patients receive only supportive care since there are no specific treatments, drugs or effective vaccines against DENV.

Dengue non-structural protein 1 (NS1) is a multifunctional glycoprotein that is essential for DENV infection. Intracellular NS1 exists as a homodimer and functions as part of the viral replication complex [6]. NS1 dimer is also found on the surface of infected cells although little is known about its function. Unlike other non-structural proteins, NS1 is secreted from infected cells as a soluble barrel-shape hexamer (sNS1), which is detectable at levels up to 50 μg/ml in the blood circulation during the onset of symptoms and persists for several days [7,8]. sNS1 can also attach back to the surface of cells via a proteoglycan interaction [9], which helps the virus to escape attack from the immune response [10–13]. NS1 is also a virulence factor associated with dengue pathogenesis since it induces endothelial membrane disruption and activates complement, platelet and immune cells [14–20]. As such, NS1 contributes to vascular leakage, inflammation, thrombocytopenia and hemorrhage [17,20–22]. Passive administration of anti-NS1 antibodies or immunization of NS1 protein protects mice from lethal DENV challenge as well as other flaviviruses [23–30]. NS1 protein vaccination has been shown to elicit the production of protective antibodies that block NS1 functions and/or activate Fc-mediated effector functions such as antibody-dependent cellular cytotoxicity (ADCC), phagocytosis, and complement-mediated lysis [24,31–34]. Therefore, NS1 is an attractive vaccine candidate because it offers protection against the virus, but does not induce ADE.

A monomeric unit of NS1 comprises three distinct domains: the β-roll (residues 1–29), the wing (residues 30–180) and the β-ladder (residues 181–352). The β-ladder has two faces, one of which is an extended β-sheet and the other which is described as a spaghetti loop [35]. The functional basis of these domains is not thoroughly understood and target epitopes for antibody-mediated protection are not comprehensive. To date, several NS1 epitopes have been identified from naturally infected humans and immunized mice [36,37], but the functions of only a few have been demonstrated. Immuno-dominant regions of the NS1 protein associated with cell binding have been identified solely on the wing and the C-terminal end of the β-ladder domains [33,38]. Protective and flavivirus cross-reactive mAbs binding to these epitopes could abrogate interactions of NS1 to the cell membrane, thereby inhibiting NS1-mediated endothelial disruption [33,38]. Moreover, these antibodies also prevent endothelial leakage caused by sNS1 from several flaviviruses because they target highly-conserved epitopes. Accordingly, broadly reactive antibodies against conserved NS1 regions or an epitope-based vaccine are considered suitable vaccine designs.

In this study, we used peripheral blood mononuclear cells (PBMCs) from subjects with secondary dengue infections (DENV1–4) to generate a dengue immune phage library displaying antibody single-chain variable fragments (scFv). scFv phages demonstrating cross-reactivity to sNS1 of all four DENV serotypes were selected and reformatted to human IgG1 mAbs. The binding activities of these mAbs to NS1 were examined by ELISA, western blot, and flow cytometry. Conserved antibody recognition regions were determined on a panel of chimeric DENV2-ZIKV NS1 antigens and by alanine mutagenesis. Two groups of DENV cross-reactive anti-NS1 mAbs that react distinctly to NS1 protein presented on the surface of DENV2-infected cells were identified. Investigation of their antigenic sites revealed that both groups recognize overlapping amino acid residues located on the N terminus of the β-ladder domain of DENV2 NS1. Germline analysis of the antibody variable genes revealed that mAbs are expressed from different B-cell germline genes of the heavy chain.

## Materials and methods

### Dengue viruses and DENV NS1 protein

Dengue viruses (DENV1 strain Hawaii, DENV2 strain 16681, DENV3 strain H87 and DENV4 strain H241) were propagated in C6/36 cells cultured in Leibovitz-15 medium (L-15, Gibco, USA) supplemented with 3% fetal bovine serum (FBS; Gibco) and 10% tryptose phosphate broth (Sigma-Aldrich, USA). The purified NS1 proteins from four dengue serotypes were obtained from cell supernatant of infected Vero cells using affinity chromatography with anti-NS1 antibody as previously described [17]. Purity of DENV NS1 was analyzed on 10% SDS-PAGE (S1 Fig).

### PBMC isolation, RNA extraction and PCR amplification of immunoglobulin variable region genes

Peripheral blood mononuclear cells (PBMCs) were isolated from heparinized blood of 12 dengue infected patients in pediatric wards at Khon Kaen and Songkhla hospitals, Thailand during 2004–2009 and kept as frozen cells. All research on humans was approved by Siriraj Institutional Review Board Protocol number 632/2559, Khon Kaen Hospital Institute Review Board in Human Research Protocol number KE60108 and ethical committee of Songkhla Hospital Protocol number 11/256. Written informed consents were obtained from all subjects.

The selected samples were collected from convalescence phases (2-week, 2-month and 6-month). All patients manifested severe dengue hemorrhagic fever (DHF) as classified by WHO 1997 criteria. Type of infection (primary or secondary infection) and dengue serotypes (DENV1–4) were confirmed by IgM/IgG captured ELISA [39] and by nested RT-PCR [40], respectively. RNA samples were extracted by RNeasy kit (QIAGEN, USA) and used as templates for cDNA synthesis (Superscript III RT kit, Invitrogen, USA). Nested PCR products of antibody variable region genes of heavy (V_H_) and light chains (V_L_) were obtained by amplification with Phusion DNA polymerase (NEB, USA) using human IgG/IgM primer set F2000 (Progen, Germany). The first round of PCR was proceeded by 1) pre-denaturation at 98°C, 30 s, followed by 2) 15 cycles of 98°C,10 s and 72°C, 15 s, and 3) final extension at 72°C for 5 min. Cloning adaptors with restriction sites were introduced at the second-round PCR using the templates from the first PCR step. The second round of PCR was proceeded by 1) pre-denaturation at 98°C, 30 s, followed by 2) 25 cycles of 98°C, 10 s and 72°C, 15 s, and 3) final extension at 72°C for 5 min.

### Generation of dengue immune scFv phage library

The V_L_ PCR products were cloned into pSEX81 phagemid (Progen) and transformed into electrocompetent *Escherichia coli* strain XL1 blue (Agilent, USA) to generate a sub-V_L_ library. Bacterial cells were cultured on 2xYT plates containing 100 μg/ml ampicillin and 1% glucose (2xYT-GA). Then, the V_H_ PCR products were sub-cloned into phagemid containing V_L_ variants. Bacterial cells harboring scFv genes were pooled and kept as a glycerol stock at -80°C. The library size was calculated from the number of colony-forming unit (cfu) after bacterial transformation with the library DNA.

To obtain scFv phages, bacterial cells containing scFv-phagemids were cultured in 2xYT-GA media until the OD_600_nm reached 0.4. Helper phage M13KO7 (NEB) was added to bacterial cultures at a multiplicity of infection equal to 1. Cells were precipitated and cultured in 2xYT media containing 100 μg/ml ampicillin, 0.1% glucose, and 50 μg/ml kanamycin (2xYT-GAK) overnight. PEG/NaCl (20% PEG 6000 and 2.5 M NaCl) solution was added to the cell culture supernatant to precipitate scFv phages. The phage pellet was re-suspended in 1xPBS and stored at -70°C. To assess the phage titers, *E*. *coli* XL-1 blue cells were infected with 10-fold serial dilutions of phages and cultured on TYE agar containing 100 μg/ml ampicillin and 1% glucose. Colonies harboring phagemids were counted and titer determined as cfu/ml. To determine the diversity of scFv variants, bacterial colonies were picked for phagemid DNA extraction using Presto^TM^ mini plasmid kit (Geneaid, Taiwan) and sequenced (Macrogen, Korea).

### Selection of DENV1–4 NS1-specific scFv phages

Two-independent biopanning experiments with scFv phages were performed with DENV NS1 protein (1 μg/ml) to obtain NS1-specific scFv phages. In the first biopanning experiment, a pool antigen of DENV1-4 NS1 was used as a bait for 2 rounds while the second biopanning used NS1 from DENV 1, 2, 3 and 4 separately and selected for 4 rounds. Phage clones demonstrating selective NS1 binding activity were screened by monoclonal phage ELISA using DENV NS1 (1μg/ml) as a target and BSA as a negative control. The NS1-bound scFv phages were detected with a 1:2500 diluted anti-M13 conjugated with HRP (GE Healthcare, USA) and assayed with tetramethylbenzidine (TMB) substrate (Thermo Fisher Scientific). The reaction was stopped by 2N H_2_SO_4_ and measured by ELISA reader at A450/620 nm. The scFv-2E11 phage was used as an anti-NS1 positive control in phage ELISA. Briefly, V_H_ and V_L_ genes for expression of mouse anti-flavivirus NS1 m2E11 mAb [41,42] were amplified and cloned into pSEX81 phagemid to construct scFv-2E11. The scFv-2E11 clone was transformed into *E*. *coli* XL1-blue and sequenced. The scFv-DHF.c phagemid, a non-NS1 binder control, was obtained during phage ELISA screening.

### Construction and expression of human anti-NS1 IgG1 mAbs

Bacterial colonies harboring scFv-phagemid were cultured in LB medium with 100 μg/ml ampicillin. Individual phagemids were extracted by Presto^TM^ mini plasmid kit and submitted for sequencing. The gene sequences were analyzed using the IMGT/V-QUEST web tool (http://www.imgt.org/IMGT_vquest). Antibody variable genes were cloned into mammalian expression vector pVITRO1_dV-IgG1/λ or pVITRO1_ dV-IgG1/κ using the polymerase incomplete primer extension (PIPE) method [43]. Specific primers to each antibody are shown in the S1 Table. The amino acid sequences of NS1 cross-reactive antibodies (V regions) are shown in the S2 Table. The antibody expression vectors were transfected into human embryonic kidney cells (HEK293T) by Lipofectamine 2000 (Invitrogen) according to the manufacturer’s protocol. After 48 h post-transfection, the cell supernatant containing antibodies was harvested. The concentration of antibodies was measured by capture ELISA. Briefly, anti-human IgG (gamma) antibody (A0423; Dako, Denmark) at a 1: 2500 dilution was coated onto wells of an ELISA plate. After washing with 0.05% PBS-Tween20 (PBST), 3% BSA was added to block non-specific protein interaction. Cell supernatant containing antibodies was diluted with 1% BSA to a 1:50 or 1:100 dilution before adding into the wells. Unbound antibody was removed by washing, and human anti-NS1 antibodies were detected by rabbit anti-human IgG (H+L) antibody conjugated to HRP at a 1: 6000 dilution (Jackson ImmunoResearch, USA). After subsequent washing to remove unbound secondary antibody, TMB was added and the reaction was stopped by 2N H_2_SO_4_. The ELISA signal was measured as OD reading at A450/620 nm.

### Western blot assay

Mock and DENV2-infected C6/36 cell lysate were prepared by RIPA buffer (Thermo Fisher Scientific) and subjected to SDS-PAGE in a non-reducing/no heat (NRNH) or a reducing/heat (RH) condition. After transfer to nitrocellulose membrane, NS1 was detected with human anti-NS1 mAbs, followed by a 1:1000 diluted rabbit anti-human IgG antibody conjugated with horseradish peroxidase (HRP) (P0214; Agilent). The immunoreactive signal was visualized by 3, 3’-diaminobenzidine (DAB) staining. A DENV1-4 NS1 cross-reactive mouse monoclonal antibody m1F11 [41], was used as a positive control, followed by rabbit anti-mouse Ig antibody conjugated with HRP (P0260; Agilent) at a 1:1000 dilution.

### NS1 ELISA

Purified NS1 (125 ng) from DENV1–4 infected Vero cells and BSA were coated onto wells of an ELISA plate at 4°C overnight. After blocking with 4% BSA, human anti-NS1 mAbs were added for 1 h at 37°C, followed by P0214 (1:5000 diluted) for another 1 h. TMB substrate was applied and the reactions were stopped by 2N H_2_SO_4_. The ELISA signal was measured by reading OD at 450/620 nm.

Purified recombinant ZIKV NS1 (500 ng) from a *Drosophila melanogaster* Schneider 2 (S2) cell line and BSA were coated onto the ELISA plate at 4˚C overnight. After blocking with 4% BSA, human anti-NS1 mAbs were added to wells of an ELISA plate. In-house anti-ZIKV NS1 ZM5 mAb was included as a positive control. The immune complexes were detected by P0214 (1:5000 diluted). TMB substrate was applied and product OD signal was measured as described above.

### Flow cytometry

Mock or DENV2-infected human immortalized hepatocyte-like cells (imHC) were used to observe intracellular and surface-exposed NS1 protein. For intracellular NS1 staining, cells were fixed and permeabilized with 2% formaldehyde and 0.1% triton X-100, respectively. Human anti-NS1 mAbs were added to the cells, followed by rabbit anti-human IgG antibody conjugated with Alexa Fluor 488 dye (Invitrogen) at a 1:500 dilution. For surface-exposed NS1 staining, live cells were detected with antibodies as described above. Propidium iodide (PI) was added immediately before flow cytometry to exclude dead cells. Flow cytometry of processed cells was performed on BD FACSCalibur™ Flow Cytometer (BD Bioscience, USA).

The binding of NS1 to cell surfaces was determined as described previously [9]. Briefly, imHC cells were removed from tissue culture plates with an EDTA solution (8mM EDTA in plain DMEM-F12). Cells (2 x 10^5^) in suspension were incubated on ice for 1 h with purified DENV2 NS1 (1 μg). After washing with serum-free medium, human anti-NS1 antibodies were added to the cells and incubated on ice for 1 h. Bound primary antibodies were detected by a 1:500 diluted goat anti-human IgG conjugated with Alexa Fluor 488 dye (Invitrogen). PI was added before detection.

### Construction and expression of recombinant DENV2, ZIKV, and chimeric DENV2-ZIKV NS1

pCAGGS-E-NS1-(Gly_4_Ser)_2_-6xHis, containing dengue natural signal sequence, DENV2 NS1 gene (DENV2 strain 16681) fused with Glycine-Serine linker and 6xHistidine tag at the C-terminus, was used as a backbone for constructing chimeric recombinant DENV2-ZIKV NS1 (rDENV2-ZIKV NS1). Ten constructs were generated by substitution of a ZIKV NS1 scaffold with DENV2 NS1 residues encompassing regions aa 1–50, aa 1–157, aa 1–177, aa 1–235, aa 1–273, aa 51–352, aa 158–352, aa 178–352, aa 236–352, and aa 274–352 (S5 Fig). pCAGGS-IgGHc-ZIKV NS1-(Gly_4_Ser)_2_-6xHis plasmid contains 19 amino acid residues of human heavy chain IgG signal sequence and ZIKV NS1 (Accession #KU681081.3). Specific primers with 15–20 bp 5’-overlapping sequence (S3 Table) were designed by online NEBuilder Assembly Tool (NEB) to generate DENV2-ZIKV NS1 chimeras or ZIKV NS1. PCR amplification was conducted by Phusion high-fidelity DNA polymerase (Thermo Fisher Scientific). Plasmids were constructed from fragments with overlapping sequences via the Gibson assembly method. The plasmids encoding recombinant NS1 (rNS1) were transfected into HEK293T cells. After 48 h post-transfection, cell supernatant containing secreted rNS1 proteins was harvested and cell lysate was prepared by RIPA buffer. Chimeric rDENV2-ZIKV NS1 proteins in cell culture supernatant were spotted onto a nitrocellulose membrane, and analyzed with human anti-NS1 mAbs and a 1:1000 diluted P0214. Expression of the rNS1 chimeras and ZIKV NS1 was confirmed by mouse anti-6xHis-tag (HIS.H8) mAb (Thermo Fisher Scientific) and polyclonal mouse anti-ZIKV NS1 antibody, respectively. The immunoreactive signal was developed by enhanced chemiluminescent substrate (ECL; Thermo Fisher Scientific). Antibody epitopes were visualized on a 3D model of DENV2 NS1 dimer (PDB:4O6B) [44] using Visual Molecular Dynamics (VMD) version 1.9.3 [45].

### Epitope mapping by alanine substitution mutagenesis

B-cell epitope prediction of surface residues on DENV2 NS1 was performed on Eillipro and Discotpe 2.0, Epitopia, and DeepviewSwiss-PdbViewer and Pymol findSurfaceResidues. The pCAGGS-E28-NS1-FL plasmid containing DENV2 NS1 (16681) gene was used as the template for alanine substitution. PCR mutagenesis was performed using specific primers (S4 Table) and the PCR products were treated with DpnI (NEB) prior to transformation into *E*. *coli* DH5-α. Single bacterial colonies were selected, extracted the plasmid DNA, and sequenced as described above. Plasmids encoding the NS1 mutants, the wild-type (wt) DENV2 NS1, and the empty vector, were transfected into imHC cells with PEI transfection reagent (Sigma-Aldrich). Cell lysates were collected and assayed by western blot with human anti-NS1 mAbs and a 1:2000 diluted goat-anti human IgG conjugated to HRP (Merck, USA). A pool of 16 mouse anti-NS1 mAbs was used as a positive antibody control. For normalization, the endogenous glyceraldehyde 3-phosphate dehydrogenase (GAPDH) protein was selected as an internal control, and detected by a 1:1000 diluted mouse anti-GAPDH antibody (Santa Cruz Biotechnology, USA). Secondary antibody P0260 was employed for detection on the mouse-derived antibodies. The immunoreactive signals were visualized by ECL. NS1 expression was quantified and normalized with GAPDH signal using ImageJ software. Percentages of normalized NS1 values were calculated and compared with that of wt NS1 (100%).

## Results

### Construction of a diverse dengue immune library representing B-cell genes of dengue infected patients and biopanning for NS1-binding scFv phage

**Fig 1:**
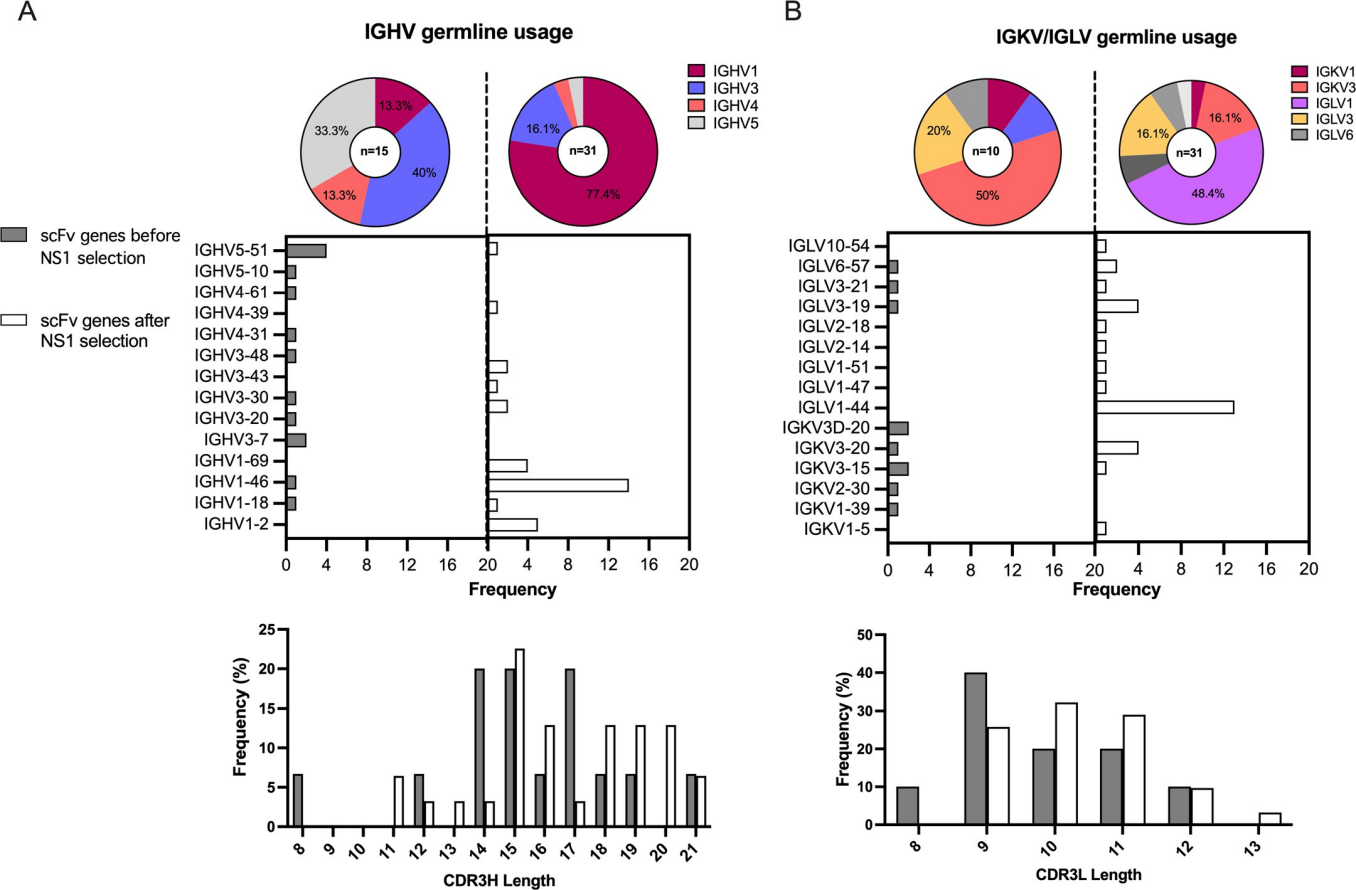
B-cell germline genes and CDR3 lengths represented among scFv clones of the (A) heavy chain and (B) light chains. The germline antibody family representation is shown in the pie charts with the total of scFv clones (top). The gene frequency of germline IGHV and IGLV/IGKV genes represented among scFv clones (middle) and the amino acid lengths of CDR3H and CDR3L (bottom) are shown in the bar graphs. The grey and white bars represent scFv clones isolated from the library before and after selection for DENV-NS1 scFv-binders, respectively.

A dengue immune phage library displaying scFv was constructed by using lymphocytes obtained during convalescent periods (2-week, 2-month, and 6-month) from 12 Thai patients who experienced secondary dengue infections (DENV1-4) and manifested severe dengue hemorrhagic fever (DHF). The size of the scFv antibody library was estimated to be 2.45 x 10^7^ clones. To determine the complexity of the library, seventeen scFv antibody library clones were randomly selected for B-cell gene sequencing. The immunoglobulin variable genes of the heavy (IGHV) and light chains (IGLV/IGKV), as well as the amino acid composition and length of the complementarity-determining region 3 (CDR3), were examined. 88% (15/17 clones) contained productive, rearranged IGHV genes, whereas two clones contained IGHV sequences from the backbone vector. The representation of IGHV1, 3, 4 and 5 gene families, heterogeneity of heavy chain CDR3 (CDR3H) amino acid sequence, and variable CDR3 length (8–21 amino acids) were noted (Fig 1A and S5 Table). The most commonly represented IGHV family was IGHV3 with six gene variants, followed by IGHV5 with five. Among the IGHV5 family variants, the IGHV5-51 gene predominated. We also examined the IGLV and IGKV genes encoding Igκ and Igλ of the light chain. Ten clones contained productive, rearranged sequences and the others showed unproductive genes due to frameshift mutations. Of the productive light chain genes, more IGKV than IGLV genes were identified, although no bias towards individual genes was apparent (Fig 1B). An assorted amino acid composition in the light chain CDR3 (CDR3L) was noted (S5 Table). The CDR3L length range (8–12 amino acids) was less than that of CDR3H (Fig 1B). Overall, the sequence data indicate that our dengue immune phage library is diverse and adequately represents antibody repertoires from dengue-infected persons.

We proceeded to isolate phages with high-affinity binding to DENV NS1 of all four serotypes from two-indepenent biopanning experiments. The antibody genes among 31 scFv phages selected for NS1-specific binders were analyzed (Fig 1A). In contrast to unselected phage, the dominant IGHV families were IGHV1 (77.4%; n = 24/31) and IGHV3 (16.1%; n = 5/31). The most represented IGHV genes were IGHV1-46 (45.2%; n = 14/31), IGHV1-2 (16.1%; n = 5/31) and IGHV1-69 (12.9%, n = 4/31). CDR3H sequences varying in length between 11–21 amino acids were identified; however, some sequences showed 80–100% identical residues suggestive of B-cell clonotypes (Table 1). We also found over-representation of IGLV (80.6%, n = 25/31) over IGKV (19.4%; n = 6/31). IGLV1 was the most common family, with IGLV1-44 the most common gene (41.9%; n = 13/31) (Fig 1B). Variable CDR3L sequences 9–13 amino acids in length were also noted (Table 1 and Fig 1B). Interestingly, pairings of the IGHV1-46 heavy chain and IGLV1-44 light chain were apparent in 10/31 clones. The clonally biased pairings are indicative of reduced complexity resulting from biopanning to select antibodies with high affinity towards DENV NS1.

**Table 1:** Immunoglobulin germline gene analysis of NS1 selective scFv phage clones.

Phage clone	IGH germline gene			CDR3H amino acids (length)	IGL/IGK germline gene		
	V-gene	J-gene	D-gene		V-gene	J-gene^a^	CDR3L amino acids
R3_NS1.C8	IGHV1-2	J6	D1-26	ARDPGLSGTYFPSYAMDV (18)	IGLV6-57	J2 or J3	QSYDSSNVV (9)
R2_NS1.G1	IGHV1-2	J6	D1-26	ARDPGLSGTYFPSYAMDV (18)	IGLV6-57	J2 or J3	QSYDGINHVV (10)
R2_NS1.G8	IGHV1-2	J6	D1-26	ARDPGLSGTYFPSYAMDV (18)	IGLV3-19	J2 or J3	NSRDSSGNHHVV (12)
R3_NS1.H2	IGHV1-2	J4	D3-10	ARGATSSGTYFMSWGFDY (18)	IGLV2-18	J1	SSYTSSSTLV (10)
R3_NS1.H8	IGHV1-2	J5	D3-3	ARAMRDFWSGHYWFDP (17)	IGKV1-5	J1	QQYNAYSWT (9)
R2_NS1.27	IGHV1-18	J5	D3-9	ARSLHDWLLYNWFDP (15)	IGKV3-20	J4	QQYGSSPST (9)
R2_NS1.6	IGHV1-46	J6	D3-10	ARDVRAGVGADGDYYHGLDV (20)	IGLV1-44	J3	AAWDDSLNGV (10)
R2_NS1.12	IGHV1-46	J6	D3-10	ARDVRAGVGADGDYYHGLDV (20)	IGLV1-44	J3	AAWDGSLSGLV (11)
R2_NS1.16	IGHV1-46	J6	D5-24	ARDRAHDYNDLPDPHAMDV (19)	IGLV1-44	J3	AAWDDSLSGPV (13)
R2_NS1.21	IGHV1-46	J6	D2-21	ARDVRAGVGADGDYYHGLDV (20)	IGLV1-44	J2 or J3	AAWDDSLAGVV (11)
R2_NS1.25	IGHV1-46	J6	D4-23	AREFVPDDDFGGTMDV (16)	IGLV1-47	J2 or J3	AAWDDSLNGVV (11)
R3_NS1.A3	IGHV1-46	J6	D3-3	AREELDDFWLYGMDV (15)	IGLV1-44	J3	ATWDDSLSAL (10)
R2_NS1.A8	IGHV1-46	J4	D3-16	ARAAVGVDLEELSLHFDF (16)	IGLV1-44	J2 or J3	ATWDDILNVV (10)
R2_NS1.B1	IGHV1-46	J5	D3-10	ARSNYYESGHYFNVDYFDP (19)	IGKV3-20	J1	QQYGSSPRT (9)
R2_NS1.C10	IGHV1-46	J6	D3-10	ARDVRAGVGADGDYYHGLDV (20)	IGLV1-44	J3	AVWDDILNGV (10)
R3_NS1.E2	IGHV1-46	J5	D3-10	ARSNYYESGHYFNVDYFDP (19)	IGKV3-20	J1	QQYGSSPRT (9)
R2_NS1.E8	IGHV1-46	J6	D2-21	ARDAGGDEDHYYMDV (15)	IGLV1-44	J1	AAWDDSLNGDV (11)
R2.NS1_F1	IGHV1-46	J6	D5-24	ARDRAHDYNDLPDPHAMDV (19)	IGKV3-20	J1	QQYGSSSWT (9)
R2_NS1.G7	IGHV1-46	J4	D3-16	ARAAVGVDLEELSLQFDF (16)	IGLV1-44	J3	AAWDDSLNGV (10)
R3_NS1.H6	IGHV1-46	J6	D2-21	ARDAGGDEDHYYMDV (15)	IGLV1-44	J3	ASWDDSLNGHV (11)
R2.NS1_14	IGHV1-69	J4	D5-24	ATSRRGRDGYRYFDY (15)	IGKV3-15	J1	QQYNNWPKT (9)
R2_NS1.31	IGHV1-69	J4	D5-24	ATSRRGRDGYRYFDY (15)	IGLV3-21	J2 or J3	QVWDSSSDHPVV (12)
R2_NS1.C4	IGHV1-69	J4	D5-24	ATSRRGRDGYRYFDY (15)	IGLV2-14	J2 or J3	SSHTSSTPVV (10)
R2_NS1.E6 (H5)	IGHV1-69	J4	D3-10	VRDSFDASGTYYDTDY (16)	IGLV1-44	J3	ATWDDTVDGVV (11)
R2_NS1.7	IGHV3-20	J6	D5-12	ARDVAVDMVATVLYHYYGMDV (21)	IGLV3-19	J3	NSRDSSGNRV (10)
R4_NS1.M2	IGHV3-20	J5	D5-12	ARDVAVDMVATVLYHYYGMDV (21)	IGLV1-44	J2 or J3	AAWDDSLNGVV (11)
R2_NS1.E4	IGHV3-30	J4	D2-21	ARDLSDSPFDY (11)	IGLV3-19	J2 or J3	NSRDSSGNHLEV (12)
R2_NS1.B5	IGHV3-43	J4	D5-12	TKGTNSLASDH (11)	IGLV3-19	J3	NSRDSSGNHLV (11)
R2.NS1_H4	IGHV3-43	J4	D1-26	VKDYGGILGLHS (12)	IGLV1-51	J3	GTWDSSLSAGV (11)
R2.NS1_4	IGHV4-39	J4	D3-22	AKSFYDMTGPFDY (13)	IGLV1-44	J3	AAWDDISGWV (10)
R2.NS1_14u	IGHV5-51	J4	D6-19	ARHLVGAGTYYFDY (14)	IGLV10-54	J2 or J3	QQYNNWPKT (9)

Table 1 (cont.) Immunoglobulin germline gene analysis of NS1 selective scFv phage clones.

^a^Alignment of J-gene among mAbs matches more than one human germline gene sequences with equal percentage of identity.

### Characterization of DENV1–4 NS1 cross-reactive scFv phages

**Fig 2:**
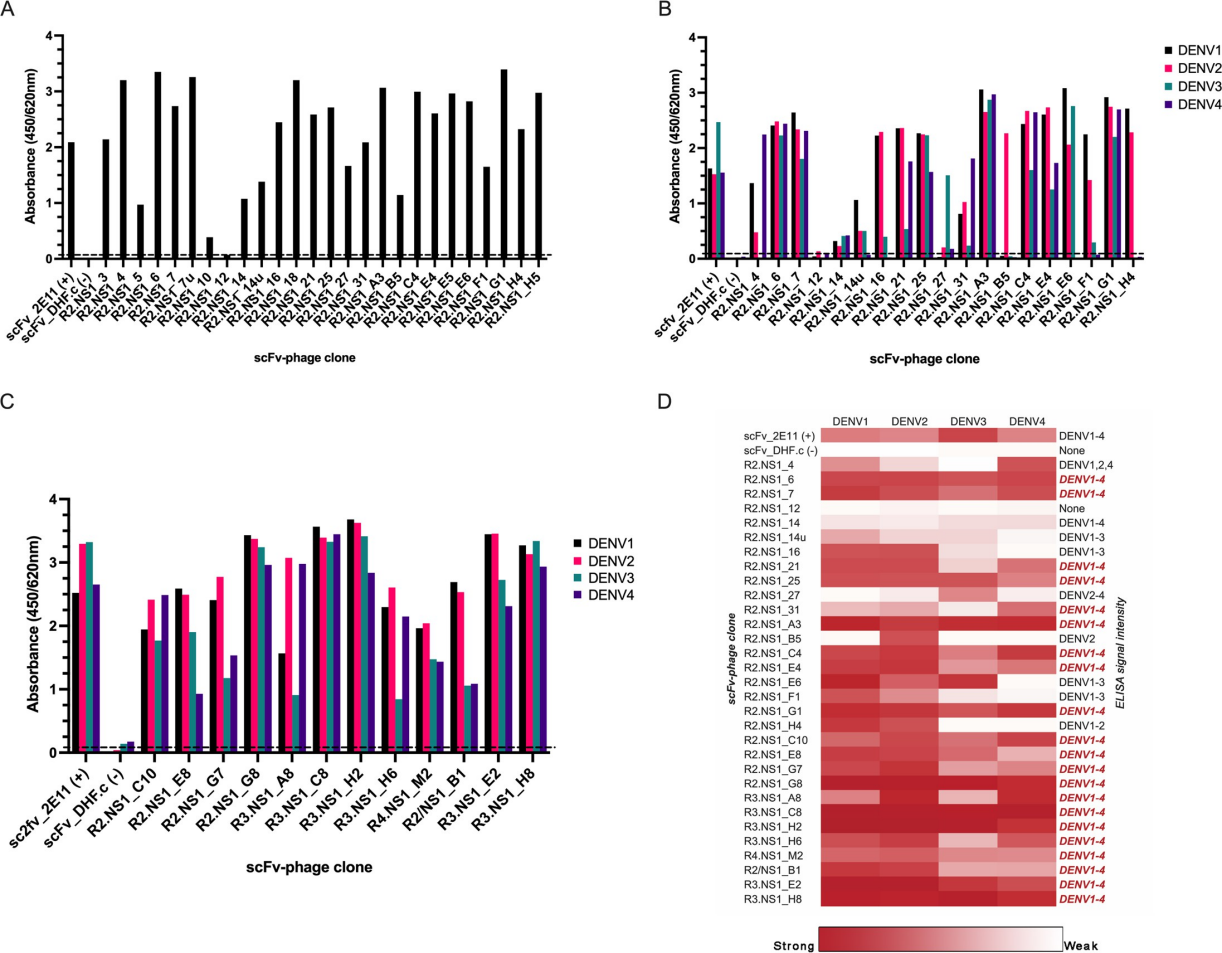
NS1-specific phage clones. (A) The reactivity of the scFv phages biopanned with pooled DENV NS1 was measured by monoclonal phage ELISA using pooled DENV1-4 NS1. (B) & (C) Selected scFv phage clones from the first (pooled DENV1-4 NS1) and second biopanning (separated NS1 from each serotype) experiments were tested with each DENV1-4 NS1 by phage ELISA. The cut-off value shown in (A-C) is OD_450/620 nm._ = 0.1. The values are from a single experiment. (D) ELISA signal intensity of the scFv phage’s binding to DENV NS1 obtained from two experiments is shown in a heat map. The spectrum of red to white indicates the range of good to no binding of scFv phages on DENV NS1.

The NS1-binding properties of selected scFv phages immunopositive for pooled DENV1-4 NS1 were initially investigated by monoclonal phage ELISA (Fig 2A). Clones R2.NS1_3 and R2.NS1_10 were not studied further owing to poor DNA sequencing results. The nucleotide sequences of clones R2.NS1_E6 and R2.NS1_H5 were identical. Another four clones with identical sequences (R2.NS1_5, R2.NS1_18, R2.NS1_7u and R2.NS1_E5) contained unproductive, rearranged IGHV sequences with a stop codon (Q6STOP) present in the framework region 1 (VH-FR1) of the antibody (S2 Fig). However, these scFv fragments could be translated from a downstream start codon in VH-FR2; moreover, their reactivity to NS1 was confirmed by ELISA (Fig 2A). As they constitute incomplete antibodies, these clones were not studied further. The reactivity of the productive scFv phage binders was tested against NS1 of each DENV serotype (Fig 2B). The majority of these scFv clones are cross-reactive to NS1 from all four DENV types. In addition to the aforementioned scFv clones, another set of DENV1-4 cross-reactive scFv clones, which show strong reactivity to NS1, was included in the subsequent experiments (Fig 2C). The ELISA signal intensity and specificity of a total scFv binders to DENV1-4 NS1 are shown as a heat map in Fig 2D. To gain further insight into broadly reactive antibodies against DENV NS1 protein, twenty-one scFv phages showing cross-reactivity to NS1 from DENV1–4, together with the R2.NS1_12 which was used as a negative control, were selected for further study.

### Phage-derived mAbs exhibit distinct NS1 binding property

**Fig 3:**
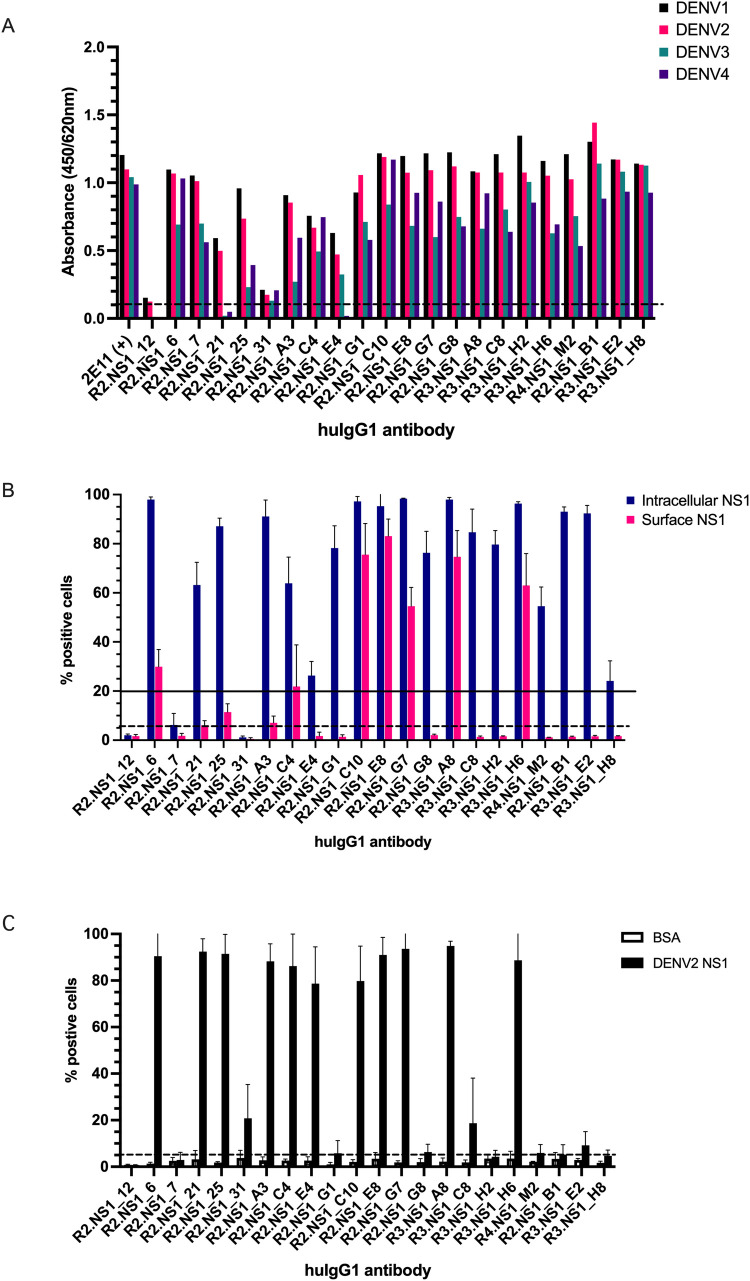
Characterization of anti-NS1 mAbs specific to DENV NS1. (A) Reformatted IgG1 antibodies were tested for NS1 serotype specificity by ELISA using each purified sNS1 derived from dengue infected Vero cells. The cut-off value is 0.1 and the values are from a single experiment. (B) Intracellular NS1 staining (blue) and surface-exposed NS1 (pink) on DENV2- infected imHC cells are shown as % positive cells (mean ± S.D.) from three independent experiments by flow cytometry. The solid line indicates the threshold for antibody binding activity (20% of positive cells) and the dot line represents the cut-off value of detection (5% of positive cells). (C) Either purified DENV2 sNS1 or BSA was incubated with uninfected imHC cells. Binding reactivity of the mAbs to binding-back NS1 was tested in three independent experiments by flow cytometry. The percentage of positive cells are represented as mean ±S.D. The dot line represents the cut-off value of detection (5% of positive cells).

To characterize the selected cross-reactive anti-NS1 antibodies, the scFv fragments were reformatted as human IgG1 antibody (mAb) and expressed in HEK293T cells. The concentrations of reformatted IgG1 mAbs in the cell culture supernatant ranged from 3 to 15 μg/ml. Cross-reactivity of the reformatted mAbs against DENV NS1 was tested by ELISA, in which sNS1 recognition of these mAbs was comparable to that of the scFv phage binders except for R2.NS1_21 and R2.NS1_E4 (Fig 3A). These two mAbs showed reduced binding to DENV3 and DENV4 NS1 after conversion to IgG1, and R2.NS1_12 mAb showed slightly increased binding toward DENV1 and DENV2 NS1. Alteration of antibody binding properties when expressed in a different format has been described previously [46]; however, we did not further investigate the discrepancies among antibodies produced in different formats.

Next, the binding properties of the mAbs on intracellular and cell surface-exposed NS1 dimers were examined on DENV2-infected imHC cells by flow cytometry. All mAbs, except R2.NS1_7 and R2.NS1_31, could detect intracellular NS1 (Fig 3B). The weak binding for R2.NS1_31 to intracellular NS1 is concordant with the weak binding observed in ELISA, whereas the results for R2.NS1_7 are discordant (Fig 3A). In contrast, most mAbs weakly reacted with surface-exposed NS1 (<20% of positive cells), with only seven mAbs showing strong binding (R2.NS1_6, C4, C10, E8, and G7; R3.NS1_A8 and H6) (Fig 3B). Since NS1 can attach back onto the cell surface, we also examined the binding property of the mAbs toward NS1 presented on the surface of uninfected imHC cells (binding-back NS1). All of the mAbs that bound to surface-exposed NS1 on DENV2-infected cells could strongly recognize binding-back NS1 (Fig 3C). Surprisingly, four mAbs (R2.NS1_21, 25, A3, and E4) reacted to binding-back NS1 but not surface-exposed NS1 on the infected cells.

### Anti-NS1 mAbs recognized the N terminal part of the β-ladder domain

We further characterized the binding regions of the 21 anti-NS1 mAbs by western blot assay. All mAbs recognized conformational NS1 epitopes since they could detect dimeric NS1 protein under the denaturing, non-reduced, no heat (NRNH) condition, but did not detect monomeric NS1 protein under the denaturing, reduced-heat (RH) condition (S3 Fig).

**Fig 4:**
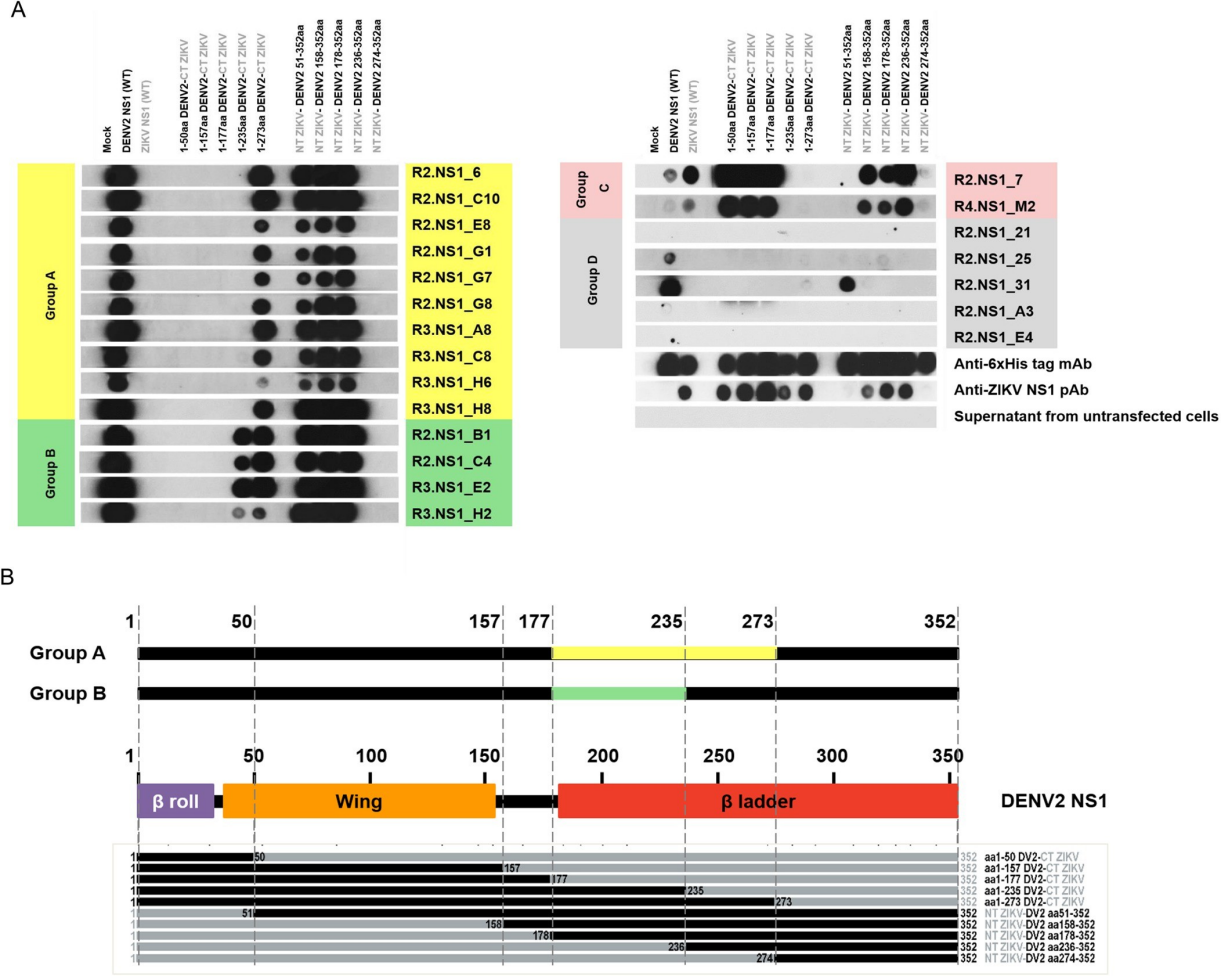
Identification of recognition sites of anti-NS1 mAbs on chimeric rDENV2-ZIKV NS1 proteins. (A) Ten soluble rDENV2-ZIKV NS1 chimeras, wt DENV2 and ZIKV NS1 were spotted onto nitrocellulose membrane and detected with different anti-NS1 mAbs. The mAbs were classified into groups A–D according to the detection patterns observed. Cell supernatant of non-transfected cells (mock) was used as the negative control. Expression of the NS1 proteins was confirmed by anti-6xHis.tag mAb and anti-ZIKV NS1 pAb. (B) The putative recognition sites of the indicated mAbs are shown with respect to the rDENV2 NS1 residues numbered from N to C-terminus with three structural domains indicated: β-roll (aa 1–29; purple), wing (aa 38–151; orange), and β-ladder (aa 181–352; red), respectively. The recognition sites of each antibody group are indicated by the colored regions (yellow, group A; green, group B).

To define the recognition sites of the mAbs, we generated a panel of chimeric recombinant NS1 proteins (rNS1) by substitution of Zika virus (ZIKV) NS1 amino acids with equivalent residues from DENV2. Most mAbs, except R2.NS1_7 and R4.NS1_M2, were minimally reactive against unmodified, wt ZIKV NS1 (S4 Fig). Ten different rDENV2-ZIKV NS1 chimeras were constructed (S5 Fig) and their expression in the cell culture supernatant was demonstrated (S6 Fig). Binding regions of the mAbs were examined on these rNS1 proteins together with wt DENV2 and ZIKV NS1 by dot blot assay (Fig 4). The mAbs were then classified based on their reactivity against rDENV2-ZIKV NS1 chimeras into four groups (A–D) as shown in the Table 2. Most mAbs belong to group A (10/21), which showed cross-reactivity to various rDENV2-ZIKV NS1 chimeras, but not wt ZIKV NS1. The mAbs in groups B and D also did not cross-react with wt ZIKV NS1, whereas the group C mAbs cross-reacted with ZIKV NS1. The group D mAbs, except R2.NS1_25 and 31, also could not react with wt DENV2 NS1, which reflects their low affinity towards rDENV2 sNS1.

**Table 2:** Characterizations of human anti-NS1 mAbs and their binding regions on DENV2 NS1.

					Binding capacity of anti-NS1 antibody specific to DENV2 NS1					Binding to chimeric DENV2-ZIKV NS1	
					NS1 ELISA^a^	WB^b^	Flow cytometry^c^			Dot blot	
No.	mAb	Isotype	Epitope type	Cross-reactivity	Hexameric NS1	Dimeric NS1	Intracellular NS1	Surface NS1	Binding back NS1	Group	AA positions
1	R2.NS1_E8	hIgG1/**λ**	conformational	DENV1-4	+++	++	95.3 ± 6.5	83.1 ± 6.9	91.0 ± 7.5	A	178–273
2	R2.NS1_C10	hIgG1/**λ**	conformational	DENV1-4	+++	+++	97.2 ± 2.0	75.6 ± 12.7	79.8 ± 15.1	A	178–273
3	R3.NS1_A8	hIgG1/**λ**	conformational	DENV1-4	+++	+++	95.3 ± 6.5	74.7 ± 10.7	94.9 ± 2.0	A	178–273
4	R3.NS1_H6	hIgG1/**λ**	conformational	DENV1-4	+++	++	96.3 ± 0.8	63.0 ± 13.0	88.7 ± 15.2	A	178–273
5	R2.NS1_G7	hIgG1/**λ**	conformational	DENV1-4	+++	++	98.3 ± 0.2	54.6 ± 7.6	91.0 ± 7.5	A	178–273
6	R2.NS1_6	hIgG1/**λ**	conformational	DENV1-4	+++	+++	98.0 ± 1.0	29.9 ± 7.0	90.5 ± 11.2	A	178–273
7	R2.NS1_G1	hIgG1/**λ**	conformational	DENV1-4	+++	+++	78.3 ± 9.0	1.4 ± 0.8	5.8 ± 5.4	A	178–273
8	R2.NS1_G8	hIgG1/**λ**	conformational	DENV1-4	+++	+++	76.3 ± 8.7	2.1 ± 0.4	6.3 ± 3.4	A	178–273
9	R3.NS1_H8	hIgG1/Ƙ	conformational	DENV1-4	+++	+++	24.1 ± 8.2	1.7 ± 0.2	4.7 ± 2.5	A	178–273
10	R3.NS1_C8	hIgG1/**λ**	conformational	DENV1-4	+++	++	84.7 ± 9.4	1.3 ± 0.4	18.7 ± 19.4	A	178–273
11	R2.NS1_B1	hIgG1/Ƙ	conformational	DENV1-4	+++	+++	93.1 ± 1.9	1.4 ± 0.3	5.2 ± 4.3	B	178–235
12	R3.NS1_E2	hIgG1/Ƙ	conformational	DENV1-4	+++	+++	92.3 ± 3.3	1.6 ± 0.3	9.2 ± 5.9	B	178–235
13	R3.NS1_H2	hIgG1/**λ**	conformational	DENV1-4	+++	++	79.7 ± 5.7	1.6 ± 0.2	4.3 ± 2.7	B	178–235
14	R2.NS1_C4	hIgG1/**λ**	conformational	DENV1-4	++	++	63.9 ± 19.7	21.9 ± 16.9	86.2 ± 13.8	B	178–235
15	R2.NS1_7	hIgG1/**λ**	conformational	DENV1-4, ZIKV	+++	+++	6.2 ± 4.7	1.8 ± 1.0	2.9 ± 3.3	C	NA
16	R4.NS1_M2	hIgG1/**λ**	conformational	DENV1-4, ZIKV	+++	+	54.6 ± 7.8	1.1 ± 0.1	5.9 ± 3.6	C	NA
17	R2.NS1_31	hIgG1/**λ**	conformational	DENV1-4	+	+	1.1 ± 0.6	0.6 ± 0.5	14.5 ± 3.3	D	158–273
18	R2.NS1_A3	hIgG1/**λ**	conformational	DENV1-4	++	+	91.1 ± 6.7	7.1 ± 2.8	88.3 ± 7.5	D	NA
19	R2.NS1_21	hIgG1/**λ**	conformational	DENV1-2	+	+	63.2 ± 9.3	6.1 ± 1.9	92.5 ± 5.5	D	NA
20	R2.NS1_25	hIgG1/**λ**	conformational	DENV1-4	++	+	87.1 ± 3.3	11.4 ± 3.4	91.4 ± 8.4	D	NA
21	R2.NS1_E4	hIgG1/**λ**	conformational	DENV1-3	+	-	26.3 ± 5.8	1.7 ± 1.6	78.7 ± 15.8	D	NA

Table 2 (cont.) Characterizations of human anti-NS1 mAbs and their binding regions on DENV2 NS1.

^a^The OD signals >1.0, 0.5–1.0, <0.5 were represented as +++, ++, +, respectively.

^b^Intensity of WB signal was shown as +++, ++, +, ranging from strong to weak binding and a negative signal was shown as -.

^c^A percentage of NS1 positive cells was obtained from 3 independent experiments and represented as mean ± S.D.

The group A mAbs recognized chimeric rNS1 proteins with DENV2 residues aa 1–273, aa 51–352, aa 158–352, and aa 178–352, suggesting that DENV2 aa 178–273 residues are essential for group A antibody recognition (Fig 4). The group B mAbs could recognize rNS1 chimeras similar to the group A mAbs; however, they also bound DENV2 residues aa 1–235, indicating that the putative mAb binding site for this group is located on DENV2 aa 178–235 residues. The group D mAb R2.NS1_31 recognized DENV2 aa 51–352, and interacted weakly with DENV2 residues aa 1–273 and aa 158–352. Therefore, DENV2 aa 158–273 residues may confer a minimal antibody binding site for this mAb. The group D mAb R2.NS1_25 showed weak immuno-positive signals for DENV2 residues aa 1–273, aa 51–352, aa 158–352 and aa 178–352; however, the signals were too low to accurately determine its binding site and we did not investigate this mAb further. We did not identify amino acid residues of the group C mAbs since these mAbs cross-react to ZIKV NS1.

### Epitope mapping using alanine mutagenesis revealed the critical binding sites of mAbs

**Fig 5:**
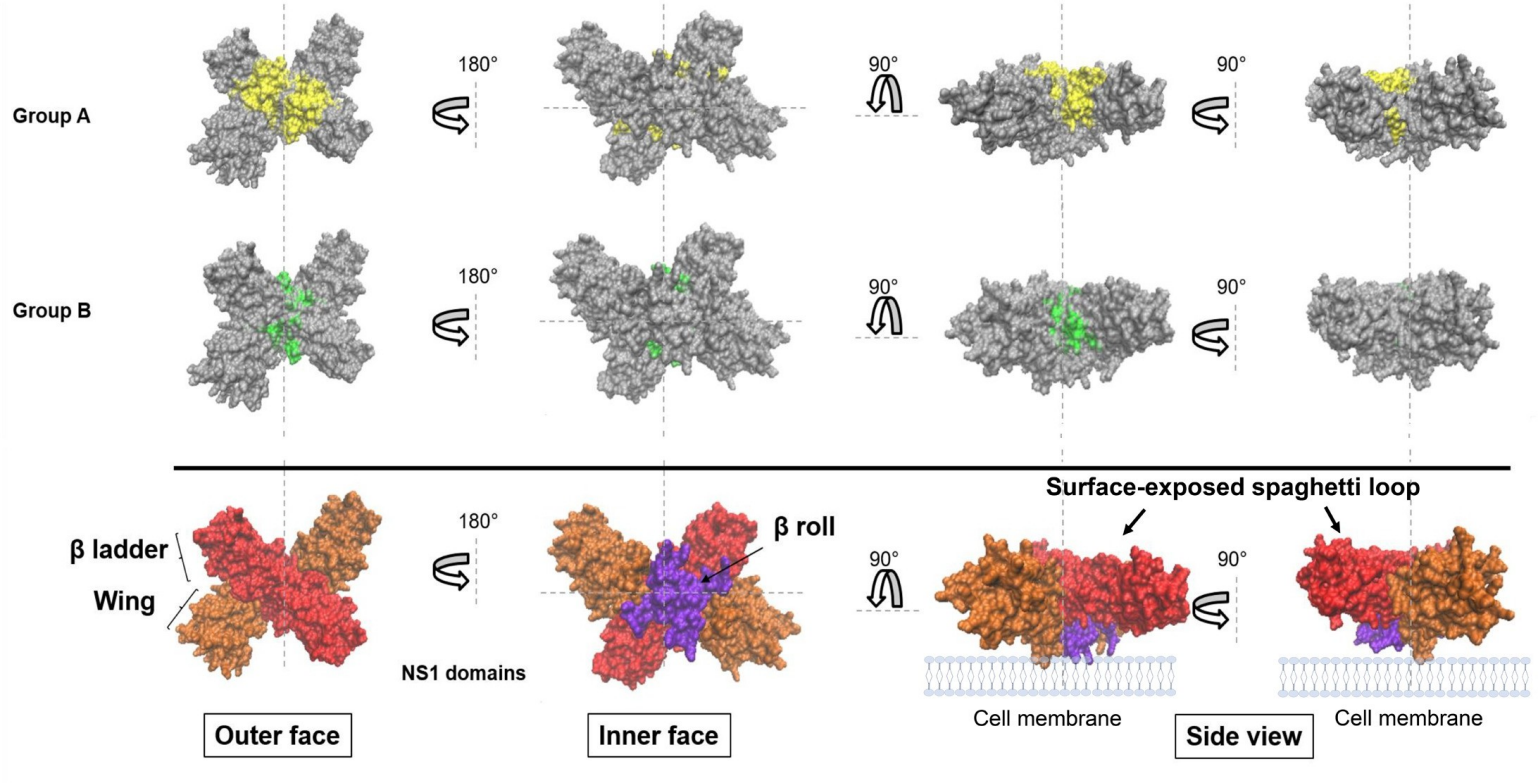
3D model illustration for the putative recognition sites on DENV2 NS1 structure. The putative binding sites for the mAbs in groups A and B are highlighted in yellow and green, respectively. The NS1 structure is shown as outer and inner faces as well as the side view. NS1 domains including the β-roll (purple), wing (orange), and β-ladder (red) are indicated in the NS1 structure.

We mapped the putative mAb recognition sites onto the 3D structure of DENV2 NS1 dimer in the group A and B mAbs (Fig 5). The putative recognition site of the group A mAbs is located on the spaghetti loop, while that of the group B mAbs is slightly shifted down towards the inner face of the NS1 structure. Considering the antibody specificity on surface-exposed NS1 on DENV infected cells (Fig 3B) and the footprint of their binding sites (Fig 4), the mAbs in the group A and B probably bind to distinct, but overlapping epitopes that are structurally conserved among all four DENV serotypes.

**Fig 6:**
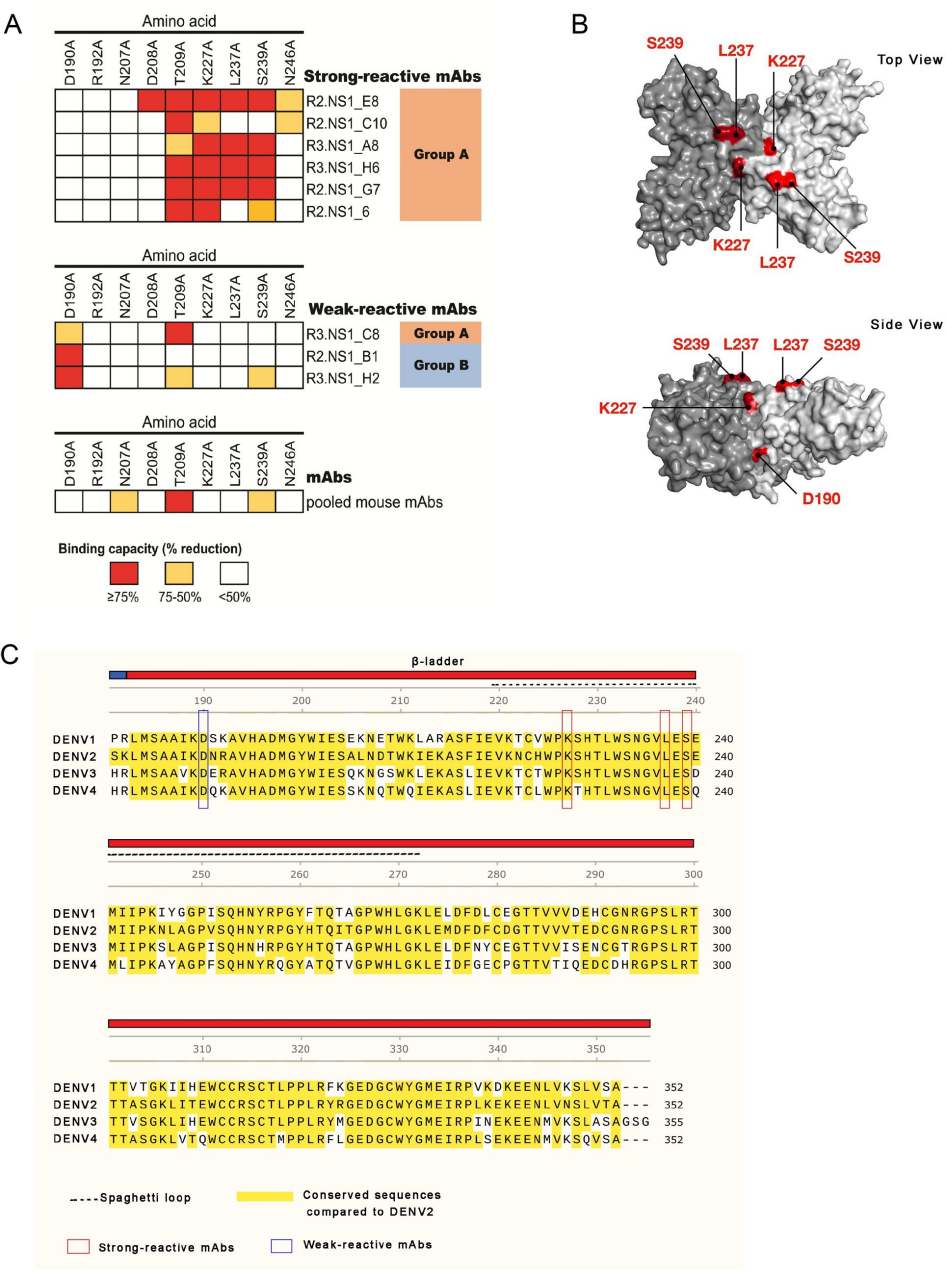
Epitope mapping of anti-NS1 mAbs by alanine substitution. Nine NS1 mutants, with predicted surface residues replaced with alanine in the positions indicated, were used to identify the critical binding sites of the strongly and weakly-reactive mAbs to surface-exposed NS1 on DENV infected cells. (A) Core and peripheral amino acid residues for the antibody recognition identified from western blot experiments (S7 Fig) are shown as red and yellow boxes, respectively. A pool of mouse anti- DENV NS1 mAbs was used as a positive control. (B) Location of core residues (marked in red) on the DENV2 dimeric NS1 structure on the top and side views. (C) Protein sequence alignment of NS1 is shown for DENV1 (Hawaii), DENV2 (16681), DENV3 (H87), and DENV4 (H241). DENV2 conserved residues are highlighted in yellow. Colored bars above the alignment indicated the β-ladder domains (aa 181–352). The dashed line indicated the surface-exposed spaghetti loop (aa 219–272). Core amino acid residues of the strong and weak reactive mAbs are indicated by the red and blue boxes, respectively.

From the location of mAb recognition sites on the NS1 structure, we hypothesized that the mAbs with strong reactivity towards cell surface-exposed NS1 bind to critical amino acid residues located on or near the surface of NS1, whereas the weakly-reactive mAbs may recognize amino acid residues located in less accessible regions. To test this hypothesis, we assessed group A and B mAbs binding to nine alanine-substituted DENV2 NS1 mutants, in which we mutated surface residues predicted by various methods (Figs 6 and S7). “Core” residues critical for mAb recognition were assigned if alanine-mutation resulted in greater than 75% loss of binding. “Peripheral” residues that are important, but perhaps not critical for mAb binding were assigned if alanine-mutation resulted in 50–75% reduction of binding. The mutants cover the N-terminal part of the β-ladder domain and the N-linked glycan motif (N-X-S/T) at N207–T209, which is required for NS1 structure and stability [47]. As expected, we found reduced binding activity on N207 and T209 mutants for several mAbs including pooled mouse monoclonal anti-NS1 antibodies. We identified three core residues, including K227, L237, and S239 among strongly-reactive mAbs in the group A (Fig 6A, top) and D190 as a core residue for weakly-reactive mAbs (Fig 6A, bottom). The core residues K227, L237, and S239 are located on the spaghetti loop, whereas D190 is located on the lateral side of NS1 (Fig 6B). All core residues are conserved among DENV NS1 serotypes (Fig 6C).

### The mAbs responsive to surface-exposed NS1 are clonally-related

Six mAbs (R2.NS1_6, C10, E8, and G7; R3.NS1_A8 and H6) that are reactive to surface-exposed NS1 on DENV2 infected cells were represented by the pairing of IGHV1-46 and IGLV1-44 genes (Table 1). This bias suggests that B cell selection and somatic hypermutation (SHM) occurred after DENV infection *in vivo*. Therefore, we suspected that the genes encoding high-affinity antibodies to DENV NS1 obtained from biopanning belong to the same clonotype group [48]. We assessed the VDJ recombination in the heavy chain and the VJ in the light chain, CDR3 amino acid composition and pairings of the heavy and light chains. A pattern of repeated VDJ repertoire usage for the IGHV1–46 gene was observed as it frequently recombined with the IGHJ genes J4 and J6, and the IGHD genes D2-21, D3-10, and D3-16 (Table 1). We identified mAbs pairs that originated from the same heavy chain gene rearrangement (R3.NS1_E8 and H6; R2.NS1_C10 and 6; R2.NS1_G7 and R3.NS1_A8 which differed by only one amino acid residue; S6 Table). For the light chain IGLV1-44 rearrangement, an overwhelming representation of IGLJ3 was noted, except for the mAb R3.NS1_E8 that used IGLJ1. These mAbs, however, presented slightly different amino acids usages in the CDR3L (Table 1).

## Discussion

NS1 is an immunoreactive molecule that is a target for both humoral and cellular immune responses. In this study, we sought to gain insight into cross-serotype DENV NS1 epitopes by generating DENV NS1 cross-reactive mAbs from a dengue immune phage library. We tested the binding properties of mAbs from phage selected by biopanning on various forms of NS1 and found that these mAbs have distinct characteristics, particularly on surface-exposed NS1. Our mapping data revealed that the core amino acid residues of the mAbs reactive to surface-exposed NS1 are located on the spaghetti loop in the β-ladder domain.

Preferential IGHV gene usage has been described in antigen-specific B cells in several viral infectious diseases including DENV infection. Various IGHV3 genes identified were likely due to the large IGHV3 family consisting of more than 50 genes, whereas the IGHV5 group has only a few members [49,50]. Moreover, the identification of the IGHV3 antibody gene was consistent with previous reports as it is the most common gene family found in DENV infection, while IGHV5-51 has been described in DENV envelope complex-epitope specific antibodies derived from memory-B cells of dengue patients [51–54]. Some IGHV genes form public clonotypes associated with many anti-viral antibodies, although these antibodies recognize distinct viral epitopes. For instance, the association of IGHV1-46 antibody with rotavirus VP6 protein [55], and IGHV1-46 and IGHV1-02 derived antibodies specific to HIV-1 gp120 [56]. The recognition of the HIV-1 envelope for these two broadly neutralizing antibodies is somewhat similar and requires interaction of the heavy chain CDR2 amino acids interface. Eliciting IGHV1-69 antibody has been widely reported in many viral infections including DENV, HIV-1, influenza A virus and H5N1 vaccination [51–54,57–59]. In addition, the biased usages of particular antibody genes could be exhibited in temporal dynamics and may be associated with disease severity. In DENV infection, the over-representation of IGHV1-02 and IGHV1-69 gene usages has been reported in acute plasmablasts of patients with dengue presenting clinical warning signs [57]. IGHV1-69 antibodies are likely to be more common in dengue patients as this antibody type has been identified in convalescence stages [51–54]. The repeated usage of this gene has been reported in DENV E and prM protein-specific antibodies, as well as polyclonal antibodies isolated from infected patients. Of interest, IGHV1-46 antibodies have not been reported to be particularly abundant in DENV infection [51,52], yet we identified DENV1-4 cross-reactive mAbs biased towards IGHV1-46 gene usage in this study.

One aspect of this work is to define DENV NS1 epitopes targeted by DENV cross-reactive anti-NS1 antibodies derived from B-cell repertoires of dengue patients. Prior studies have identified several immunodominant epitopes located on the wing and C-terminal half of the β-ladder domains [37]. Biering et.al. reported that flavivirus NS1 cross-reactive mAb (m2B7) can block NS1-mediated endothelial dysfunction; this mAb recognizes an epitope located in NS1 aa 281–327 [33]. Another cross-reactive anti-NS1 mAb, m1G5, which inhibits endothelial cell permeability, targets NS1 at aa 339–345 [38]. Furthermore, antibody profiles of human subjects with natural DENV infections have revealed the dominant epitopes located on the wing (aa 101–135) and β-ladder (aa 296–335) domains [36]. DENV2 NS1 vaccination in mice that protects against lethal DENV challenge also generates cross-reactive antibody responses, and the antibody targets span the β-roll (aa 21–40), wing (aa 101–135) and β-ladder domains (aa 156–175, aa 231–255 and aa 296–335) [36]. Interestingly, our mAbs reactive to surface-exposed NS1 on DENV2 infected cells (the group A mAbs) recognize the same amino acid residues (K227, L237, and S239) located on the epitopes identified in the vaccinated mice (aa 231–255). Further *in vivo* experiments should be performed to investigate the protective function of these mAbs.

Immunodominant epitopes around the wing and β-ladder domains are likely recognized by antibodies responsive to both natural infection and vaccination [33,36–38]. High antibody responses to NS1 epitope at the β-ladder domain (aa 341–353) are observed in sera of DF patients with secondary acute infection when compared to DHF patients, suggesting that epitopes located at C-terminus of NS1 protein may associate to protection [60]. However, some NS1 antibodies can recognize cross-reactive epitopes to human proteins and cells; most of them are identified in the wing and β-ladder domains. Epitopes located at the wing domain (aa 116–119) resembles to human LYRIC protein and a recognition of antibodies to this protein is associated with endothelial cell damage [18,61]. Epitopes located on the β-ladder domain (aa 305–330) are also cross-reactive to human fibrinogen, thrombocytes, platelet and endothelial cells, and antibody binding to their targets can produce hemorrhage in a mouse model [62,63]. Antibody responses to these proximal epitopes around the wing and β-ladder domains can be either protective or harmful.

The distinctive properties of anti-NS1 mAbs obtained in this study suggest that the antibodies play different roles in dengue pathogenesis. On one hand, the mAbs targeting NS1 epitopes on the infected cell’s surface might activate Fc receptor-mediated effector functions such as complement, phagocytosis and ADCC and these mechanisms could help clearance of DENV infected cells [27,32,64]. On the other hand, these antibodies might also bind NS1 localized on the surface of non-infected cells and damage these cells. Antibodies that recognize sNS1 might inhibit the ability of sNS1 to attach back to the cell surface and the immune complex could be cleared from the circulation. Hence, these antibodies could also be protective. However, excessive immune activation might induce massive cytokine and chemokine production, which exacerbate the disease severity. Since the balance of immune activation through anti-NS1 antibody in DENV infection remains unclear, the mechanisms and roles of NS1 cross-reactive antibodies need to be further examined.

Although the key determinants of mAb epitope recognition were identified for most of the selected mAbs, some caveats apply. For the rDENV2-ZIKV NS1 chimeras, we measured the expression of these proteins by dot-blot assay as they were not purified from the HEK293T cell supernatant. Variation in the expression level of the chimeric proteins may affect the ability to detect binding of particular mAbs that have a weak binding affinity. In addition, the use of these chimeric proteins is limited for testing on DENV2 NS1 and could not be used for the mAbs that are cross-reactive to ZIKV NS1 as seen in the group C mAbs (Fig 4). Moreover, the fine epitope mapping by alanine substitution was not comprehensive and does not suffice to predict the key residues for some mAbs. Mapping methodology may be improved by using charge-reversal substitution instead. Structure elucidation of interaction between NS1 and anti-NS1 mAbs by X-ray crystallization or cryo-EM would be useful to identify binding epitopes.

In summary, we identified DENV NS1 cross-reactive antibodies that exhibit distinct NS1 binding properties. From the epitope mapping experiments, we demonstrated that these mAbs target overlapping epitopes residing on the N-terminal part of the β-ladder domain of DENV NS1. This information could point the way towards the development of an epitope-based vaccine that targets conserved sites on surface-exposed NS1 protein expressed from DENV infected cells.

## Supporting information

S1 Fig Coomassie-stained SDS-PAGE showing purified DENV NS1 protein. DENV1-4 NS1 were prepared from cell supernatant of DENV-infected Vero cells and purified by affinity chromatography with anti-NS1 antibody. 500 ng NS1 of each serotype was analyzed by 10% SDS-PAGE under a denaturing, non-reduced/no heat (NRNH) condition and Coomassie brilliant blue R-250 staining.
(TIF)

S2 Fig Organization of the unproductive rearranged IGHV sequences. A stop codon was detected in FR1 of the VH gene. The incomplete scFv sequences could be translated from a downstream start codon at the end of FR2 (open reading frame indicated as a yellow arrow). The illustration was drawn in Snapgene. RBS: ribosome binding site; Cys: Cysteine; CDR: Complementarity-determining regions; VL: V gene of light chain; AA: amino acid at CDR3 on VH (heavy chain).
(TIF)

S3 Fig Detection of NS1 by western blot assay. Either dimeric or monomeric NS1 derived from DENV2 infected C6/36 cell lysate was examined in the non-reduced/no heat (NRNH; top panel) or reduced/heat (RH; bottom panel) conditions, respectively. Immunoblots were assayed with each anti-NS1 mAb and then detected by anti-human immunoglobulins conjugated with HRP (1:2000). Immunoblot signal was visualized by 3, 3’-diaminobenzidine (DAB) staining. The dimeric and monomeric NS1 migrate at approximately 70 and 40 kDa, respectively. Mouse 1F11 anti-NS1 mAb, which binds to both NS1 forms, was used as a positive control.
(TIF)

S4 Fig Antibody cross-reactivity to ZIKV NS1 by ELISA. 21 anti-NS1 mAbs were assayed against 500 ng of purified rZIKV NS1. The reactivity of the mAbs was detected by anti-human IgG-HRP used at a dilution of 1:5000. The signal was developed with TMB substrate and measured OD reading by ELISA reader at A450/620 nm. Anti-ZIKV NS1 (ZM5 mAb) and mock cell supernatant were included as positive and negative controls, respectively. The values shown are from a single experiment.
(TIF)

S5 Fig Plasmid constructs for expression of chimeric DENV2-ZIKV NS1 proteins. (A) Schematic picture of DENV2 NS1 protein sequences numbered from N to C-terminus, with structural domains indicated: β-roll (aa 1–29; purple), wing (aa 38–151; orange), and β-ladder (aa 181–352; red), respectively. The NS1 regions of DENV2 (black) and ZIKV (grey) are indicated for the rDENV2-ZIKV NS1 chimeras. (B) The cloning process of the rDENV2-ZIKV NS1 proteins. Truncated gene fragments of DENV2 and ZIKV NS1 were generated. The homologous overlapping regions from both DENV2 and ZIKV were used to generate full-length chimeric NS1 proteins by the Gibson assembly technique. These rNS1 proteins were fused with dengue natural secretory signal sequence (E-Sig) and 6xHistidine tag (6xHis) at N- and C-termini, respectively.
(TIF)

S6 Fig Expression of rDENV2-ZIKV NS1 proteins in HEK293T cell lysate and supernatant. Expression and secretion of rNS1 proteins were confirmed by anti-6xHis tag mAb, anti-DENV NS1 mAbs recognizing either the wing domain (m2E11), and the C-terminal part of DENV2 NS1 sequences (m2G6), as well as polyclonal anti-ZIKV NS1 pAb. Mock supernatant, which is devoid of rNS1 protein, was used as the negative control.
(TIF)

S7 Fig Detection of NS1 epitopes by western blot assay. (A) Alanine-substituted NS1 proteins were subjected to SDS-PAGE and transferred to nitrocellulose membrane. Mutant NS1 proteins, wt DENV2 NS1 (NS1) and empty vector (mock) were detected by strong and weak-reactive mAbs in the groups A and B, followed by goat anti-human IgGs conjugated with HRP (1:4000). Immuno-reactive activity was visualized by ECL. Pooled mouse anti-NS1 mAb was used as a positive control. GAPDH was used as an internal control and detected by mouse anti-GAPDH antibody (1:1000), followed by P0260. (B) NS1 protein expression was quantified and normalized with GAPDH signal. The percentages of normalized values from a single experiment were plotted. wt DENV2 NS1 (NS1) and mock were set as 100 and 0%, respectively.
(TIF)

S1 Table Primer set for scFv phage construction. (XLSX)

S2 Table Complete amino acid sequences of V_H_ and V_L_ regions of 21 anti-NS1 mAbs. (XLSX)

S3 Table Primer set for the rDENV2-ZIKV NS1 chimeras. (XLSX)

S4 Table Primers for alanine mutagenesis. (XLSX)

S5 Table Immunoglobulin germline gene analysis of scFv library phage clones before NS1 selection. (XLSX)

S6 Table CDR3H and CDR3L amino acid sequences of the strong-reactive mAbs. (XLSX)

S1 Raw images (PDF)
