# Supplementary figures and images for: Cross-reactive antibodies targeting surface-exposed non-structural protein 1 (NS1) of dengue virus-infected cells recognize epitopes on the spaghetti loop of the β-ladder domain
Source: PLoS One. 2022 May 26;17(5):e0266136. doi: 10.1371/journal.pone.0266136 (PMC9135231; doi:10.1371/journal.pone.0266136)

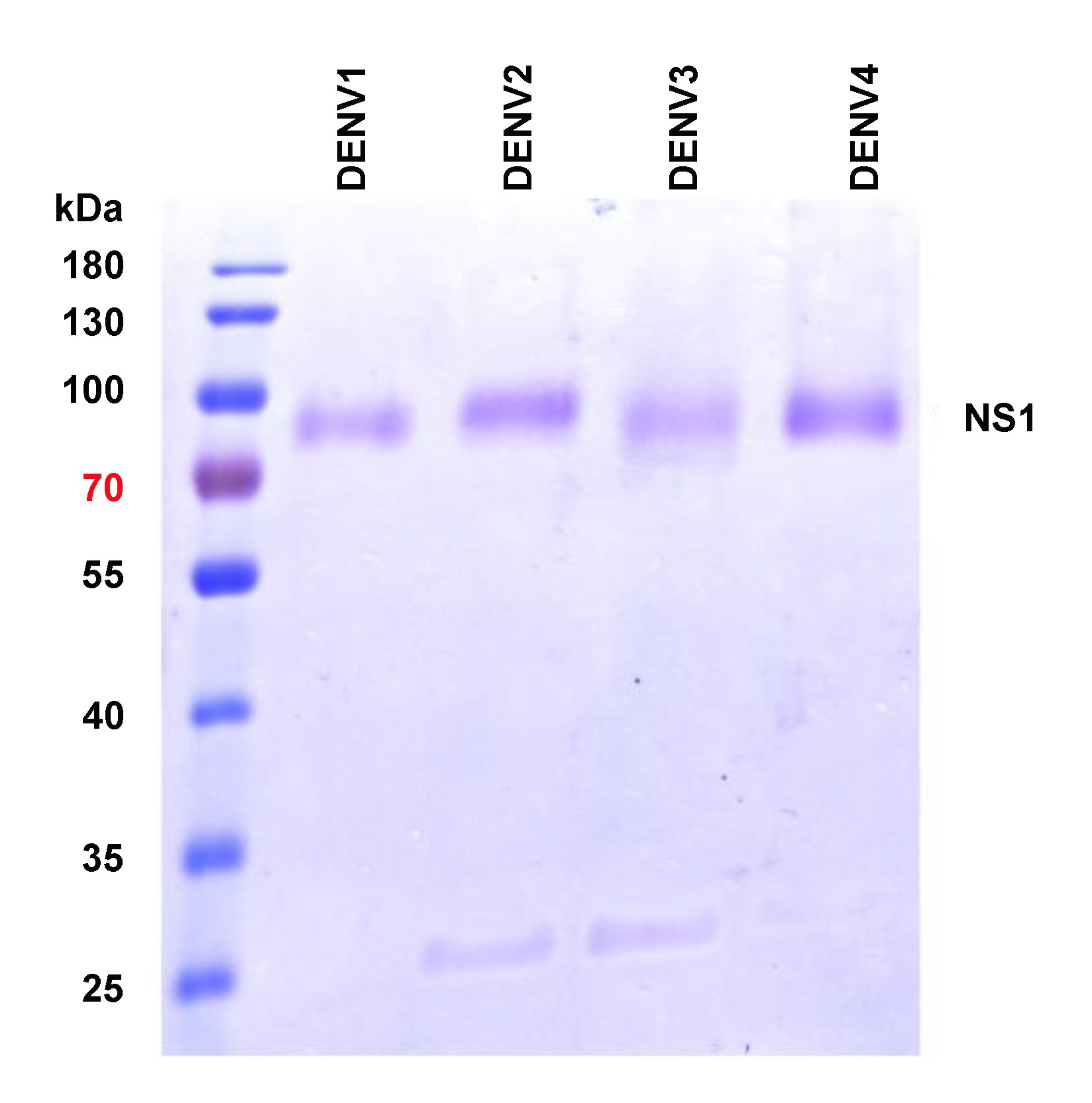

Supplement: S1 Fig — DENV1-4 NS1 were prepared from cell supernatant of DENV-infected Vero cells and purified by affinity chromatography with anti-NS1 antibody. 500 ng NS1 of each serotype was analyzed by 10% SDS-PAGE under a denaturing, non-reduced/no heat (NRNH) condition and Coomassie brilliant blue R-250 staining. (TIF) [file pone.0266136.s001.tif]

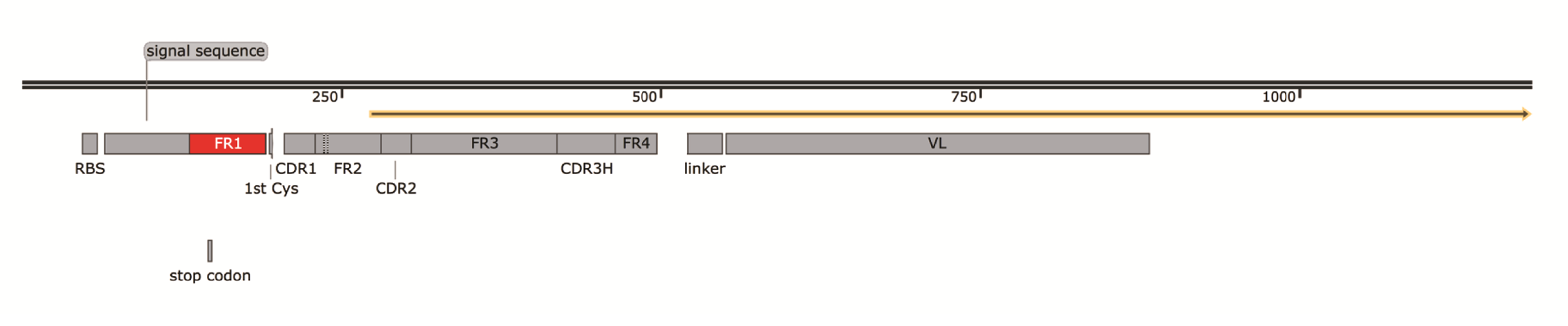

Supplement: S2 Fig — A stop codon was detected in FR1 of the VH gene. The incomplete scFv sequences could be translated from a downstream start codon at the end of FR2 (open reading frame indicated as a yellow arrow). The illustration was drawn in Snapgene. RBS: ribosome binding site; Cys: Cysteine; CDR: Complementarity-determining regions; VL: V gene of light chain; AA: amino acid at CDR3 on VH (heavy chain). (TIF) [file pone.0266136.s002.tif]

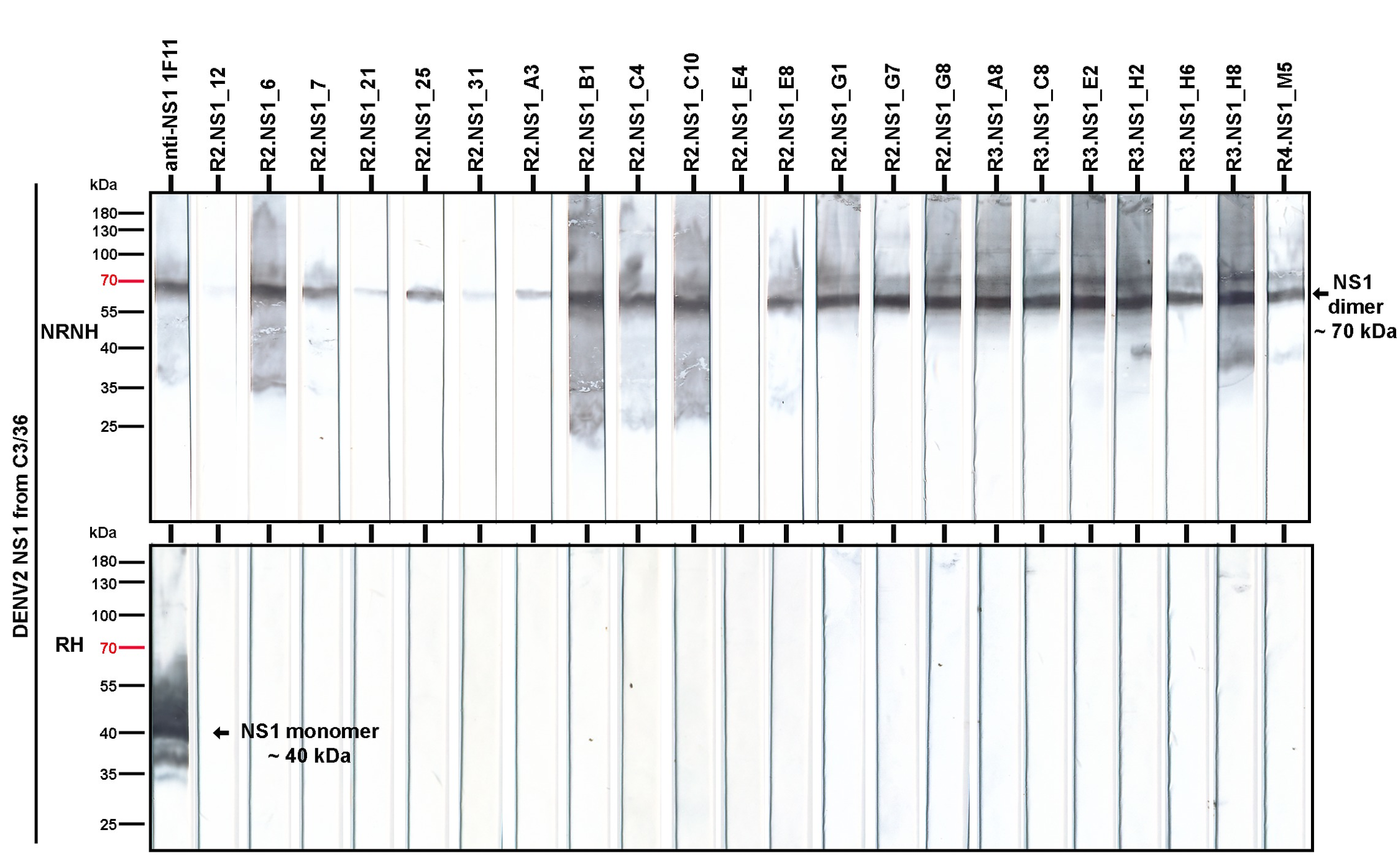

Supplement: S3 Fig — Either dimeric or monomeric NS1 derived from DENV2 infected C6/36 cell lysate was examined in the non-reduced/no heat (NRNH; top panel) or reduced/heat (RH; bottom panel) conditions, respectively. Immunoblots were assayed with each anti-NS1 mAb and then detected by anti-human immunoglobulins conjugated with HRP (1:2000). Immunoblot signal was visualized by 3, 3’-diaminobenzidine (DAB) staining. The dimeric and monomeric NS1 migrate at approximately 70 and 40 kDa, respectively. Mouse 1F11 anti-NS1 mAb, which binds to both NS1 forms, was used as a positive control. (TIF) [file pone.0266136.s003.tif]

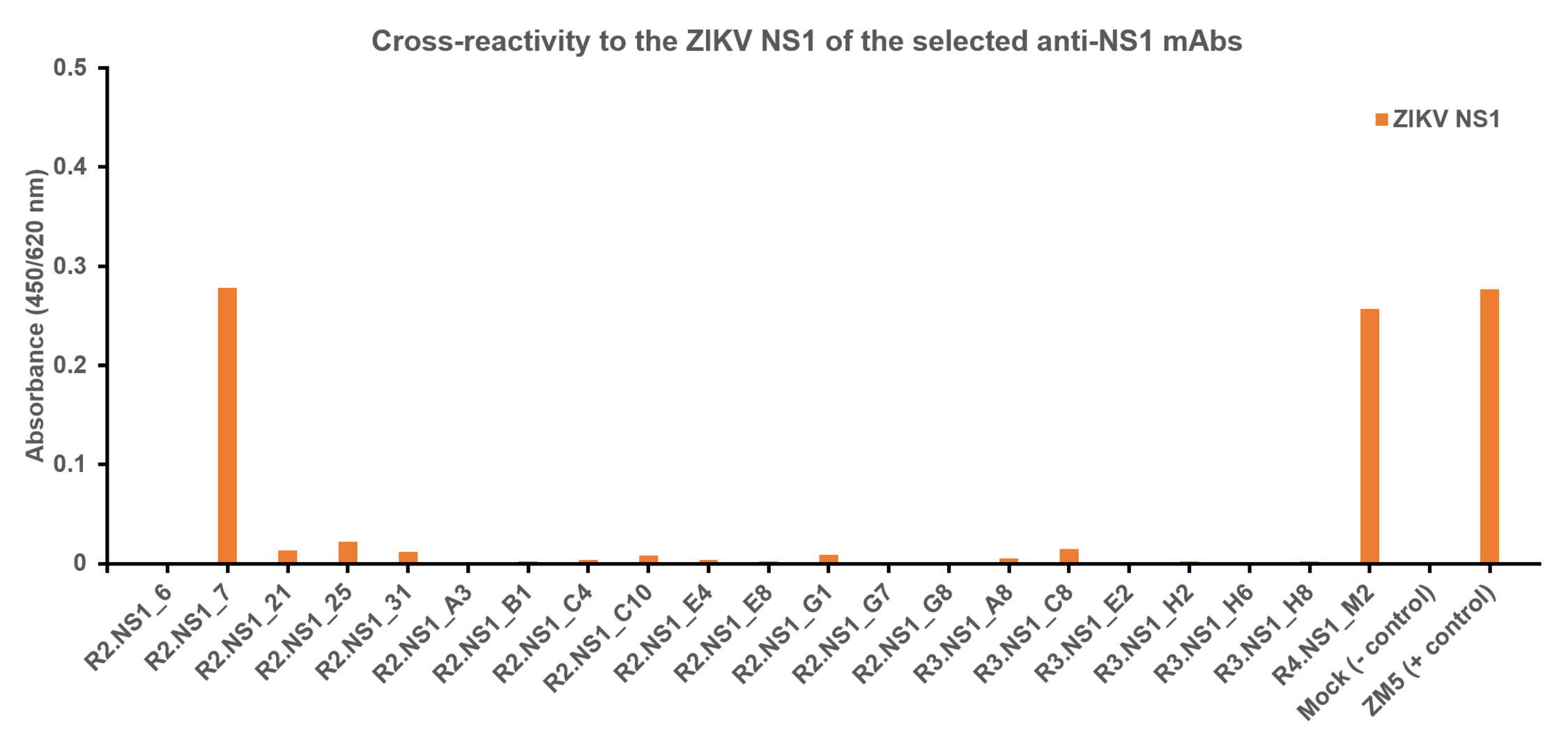

Supplement: S4 Fig — 21 anti-NS1 mAbs were assayed against 500 ng of purified rZIKV NS1. The reactivity of the mAbs was detected by anti-human IgG-HRP used at a dilution of 1:5000. The signal was developed with TMB substrate and measured OD reading by ELISA reader at A450/620 nm. Anti-ZIKV NS1 (ZM5 mAb) and mock cell supernatant were included as positive and negative controls, respectively. The values shown are from a single experiment. (TIF) [file pone.0266136.s004.tif]

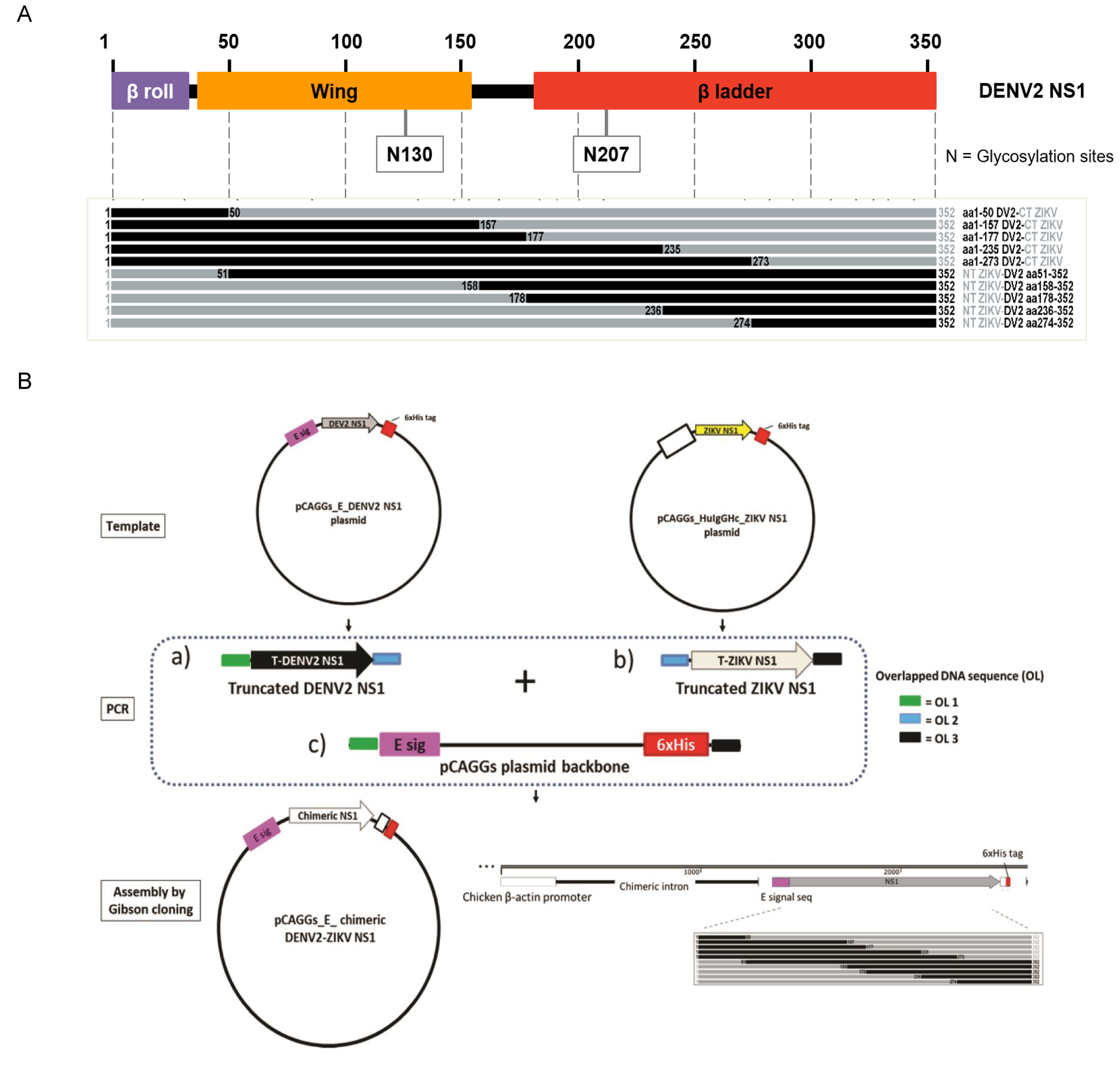

Supplement: S5 Fig — (A) Schematic picture of DENV2 NS1 protein sequences numbered from N to C-terminus, with structural domains indicated: β-roll (aa 1–29; purple), wing (aa 38–151; orange), and β-ladder (aa 181–352; red), respectively. The NS1 regions of DENV2 (black) and ZIKV (grey) are indicated for the rDENV2-ZIKV NS1 chimeras. (B) The cloning process of the rDENV2-ZIKV NS1 proteins. Truncated gene fragments of DENV2 and ZIKV NS1 were generated. The homologous overlapping regions from both DENV2 and ZIKV were used to generate full-length chimeric NS1 proteins by the Gibson assembly technique. These rNS1 proteins were fused with dengue natural secretory signal sequence (E-Sig) and 6xHistidine tag (6xHis) at N- and C-termini, respectively. (TIF) [file pone.0266136.s005.tif]

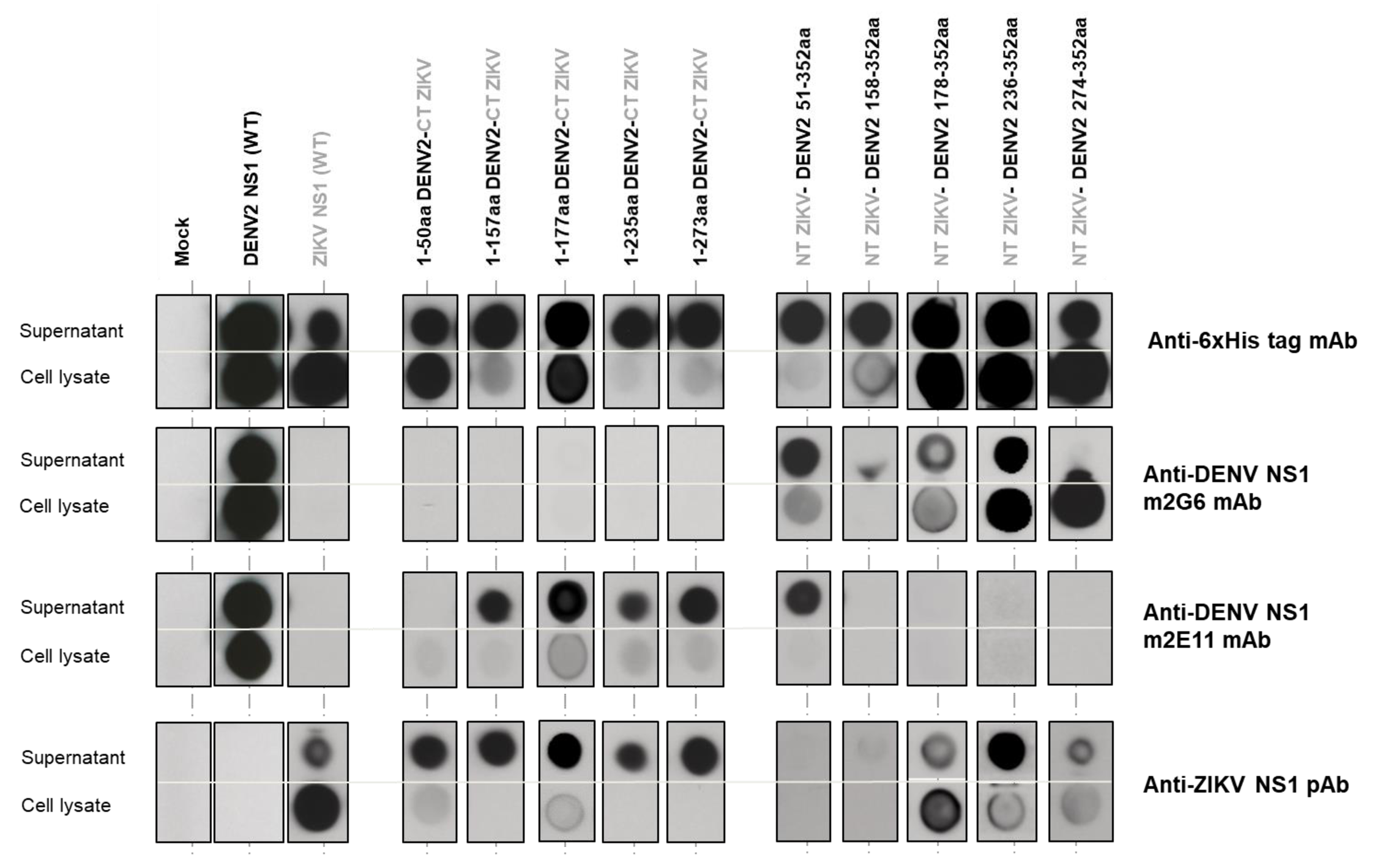

Supplement: S6 Fig — Expression and secretion of rNS1 proteins were confirmed by anti-6xHis tag mAb, anti-DENV NS1 mAbs recognizing either the wing domain (m2E11), and the C-terminal part of DENV2 NS1 sequences (m2G6), as well as polyclonal anti-ZIKV NS1 pAb. Mock supernatant, which is devoid of rNS1 protein, was used as the negative control. (TIF) [file pone.0266136.s006.tif]

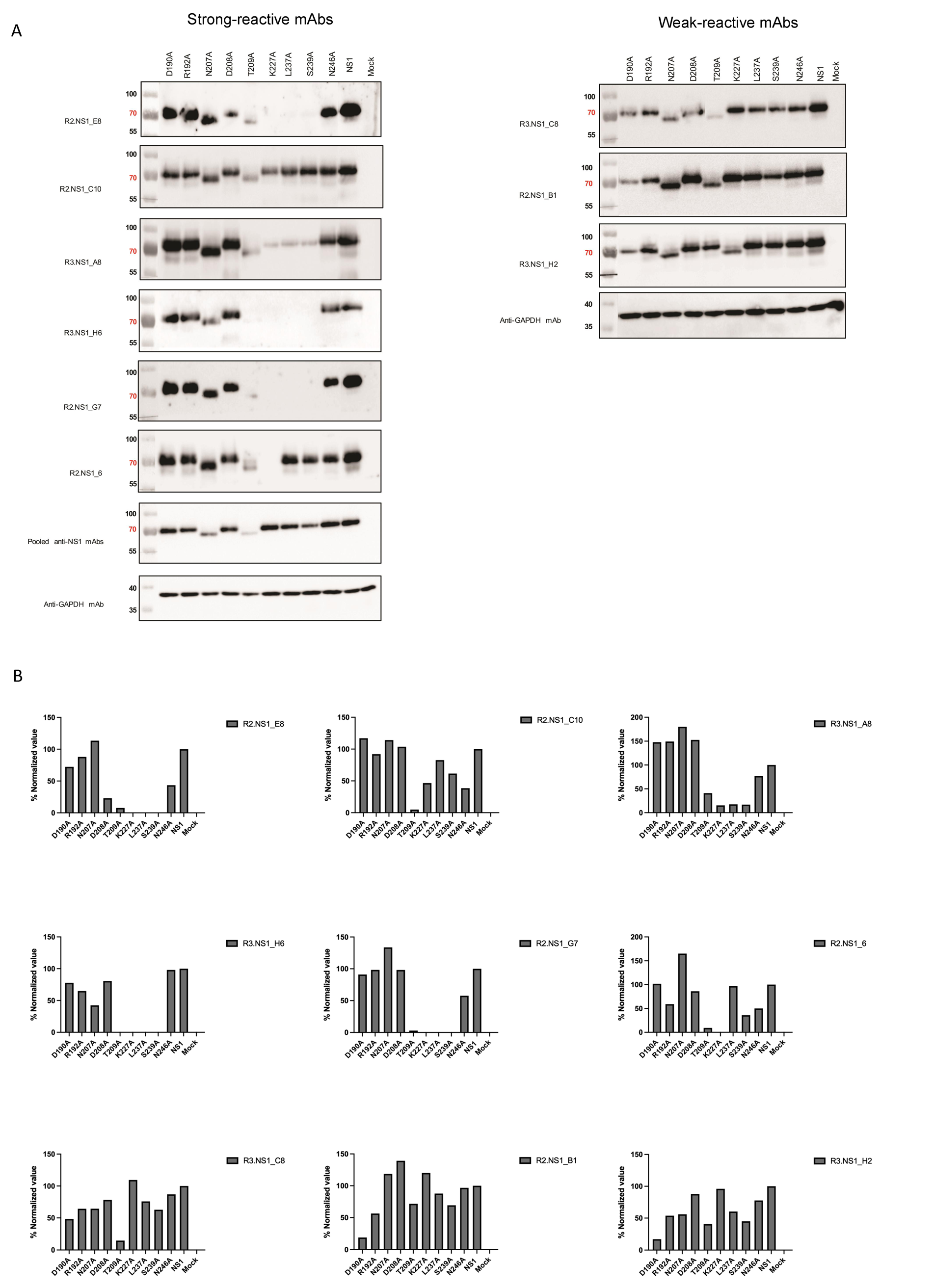

Supplement: S7 Fig — (A) Alanine-substituted NS1 proteins were subjected to SDS-PAGE and transferred to nitrocellulose membrane. Mutant NS1 proteins, wt DENV2 NS1 (NS1) and empty vector (mock) were detected by strong and weak-reactive mAbs in the groups A and B, followed by goat anti-human IgGs conjugated with HRP (1:4000). Immuno-reactive activity was visualized by ECL. Pooled mouse anti-NS1 mAb was used as a positive control. GAPDH was used as an internal control and detected by mouse anti-GAPDH antibody (1:1000), followed by P0260. (B) NS1 protein expression was quantified and normalized with GAPDH signal. The percentages of normalized values from a single experiment were plotted. wt DENV2 NS1 (NS1) and mock were set as 100 and 0%, respectively. (TIF) [file pone.0266136.s007.tif]
