# Supplementary material for: Cross-reactive antibodies targeting surface-exposed non-structural protein 1 (NS1) of dengue virus-infected cells recognize epitopes on the spaghetti loop of the β-ladder domain
Source: PLoS One. 2022 May 26;17(5):e0266136. doi: 10.1371/journal.pone.0266136 (PMC9135231; doi:10.1371/journal.pone.0266136)
Supplement: S1 Raw images — (PDF) [file pone.0266136.s014.pdf]

Raw images for Fig 4A

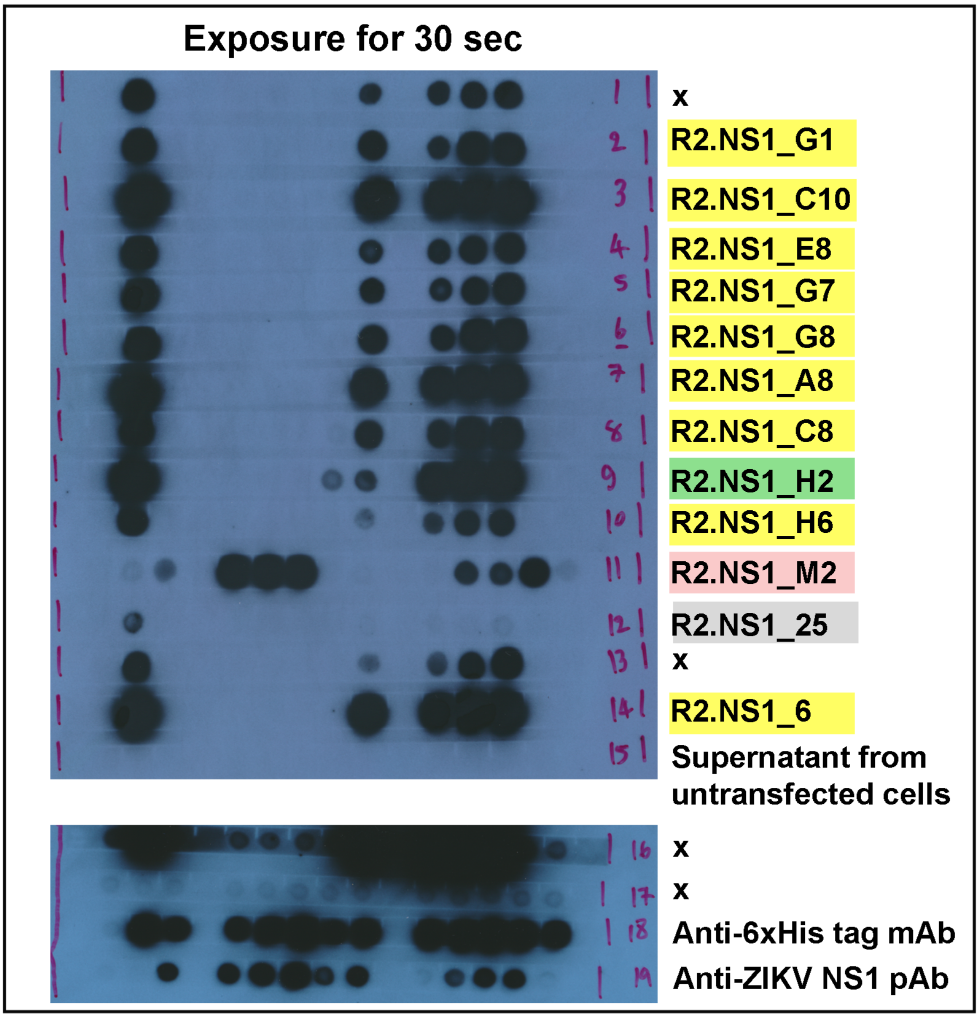

Original dot blot images in respecting to each mAb in Fig 4A epitope mapping are indicated.

Colors represented mAb in group A, B, C, and D are highlighted in yellow, green, pink, and grey, respectively.

Blot signals were obtained by film exposure at time indicated above.

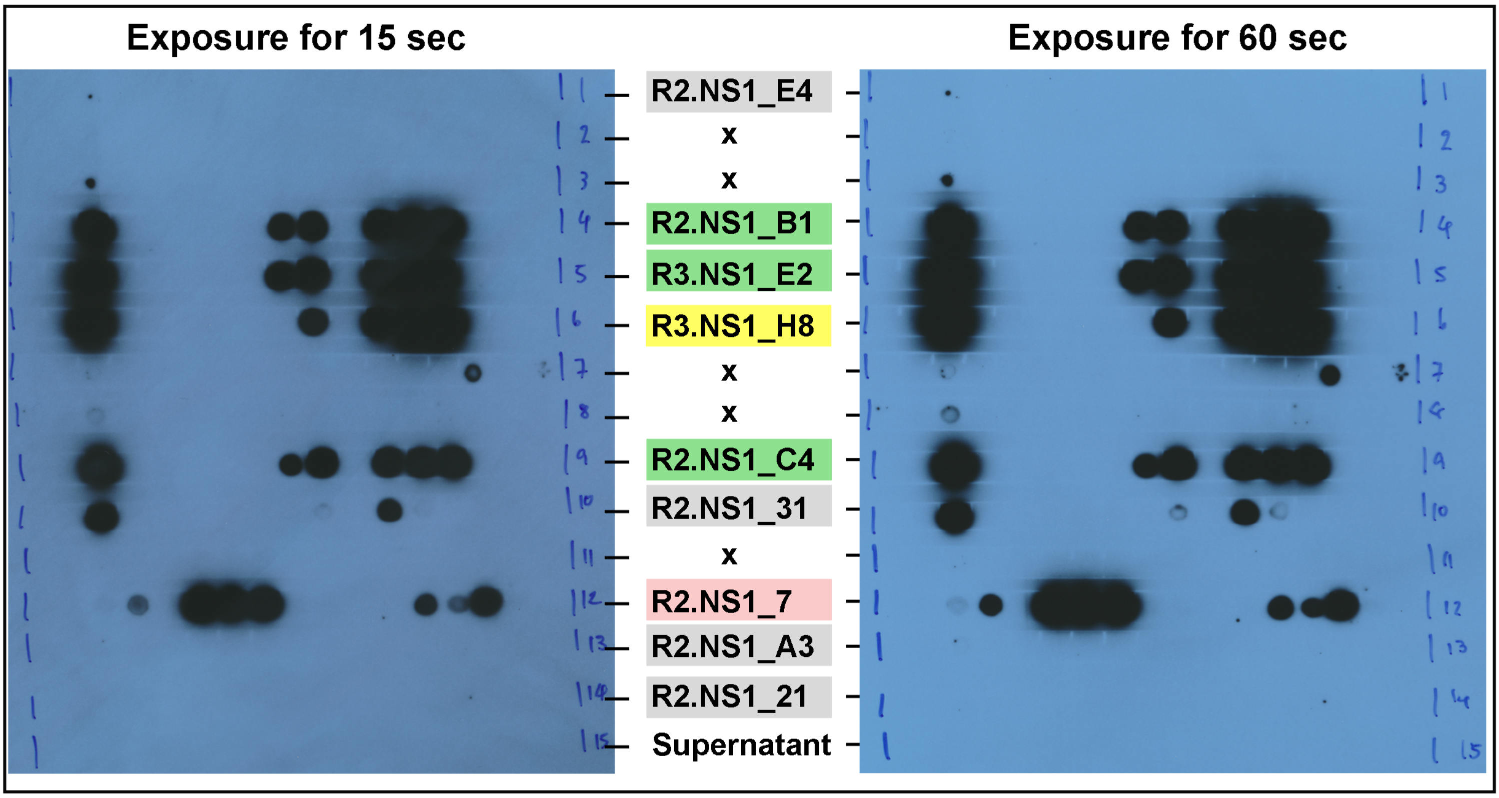

Raw images for S3 Fig.

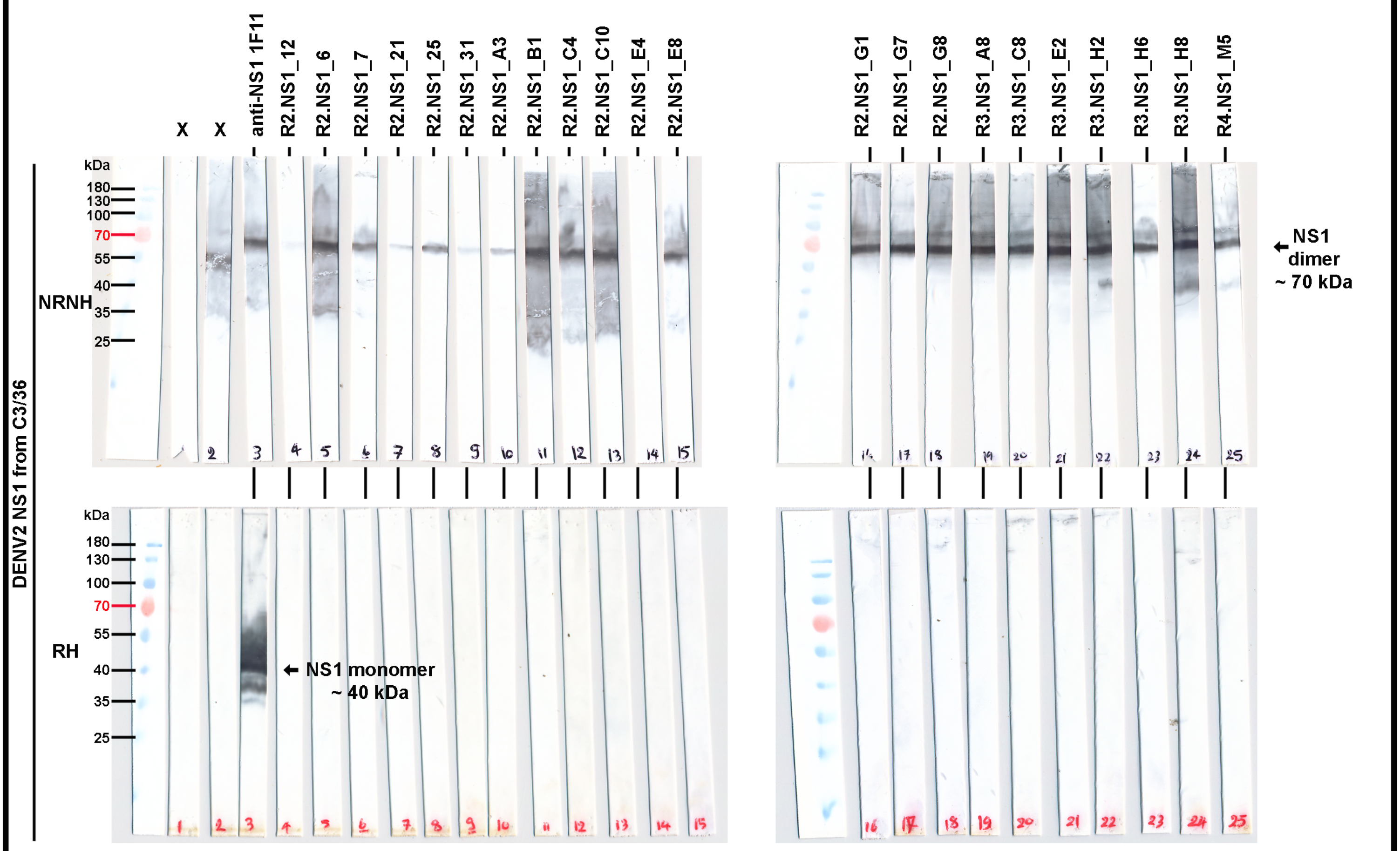

Charaterization on the binding of 21 anti-NS1 mAbs against DENV2 NS1 from infected C6/36 cell lysate by western blot. DENV2 NS1 the cell lysate on all blots (NRNH and RH) was from the same lot. Anti-NS1 mAbs from HEK293T cell supernatant were analyzed and detected by anti-human immunoglobulins conjugated with HRP (1:2000). Immunoblot signal was visualized by ECL substrate and 3, 3'-diaminobenzidine (DAB) staining. The dimeric and monomeric NS1 migrate at approximately 70 and 40 kDa, respectively. Mouse 1F11 anti-NS1 mAb, which binds to both NS1 oligomers, was used as a positive control.

Raw images for S6 Fig.

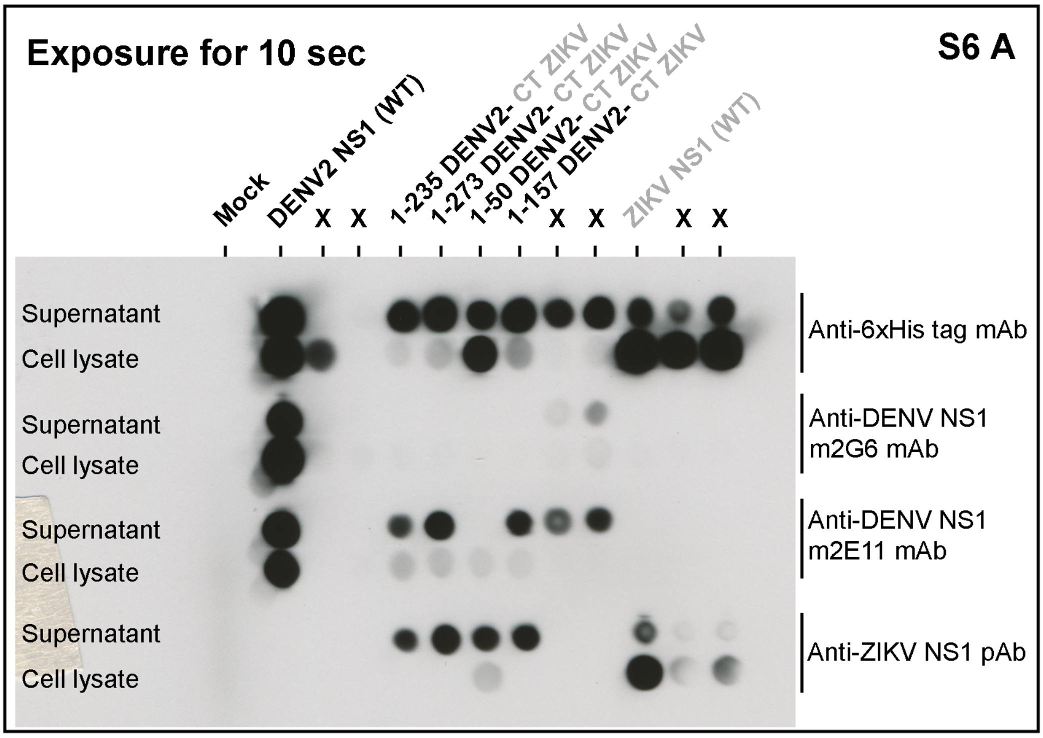

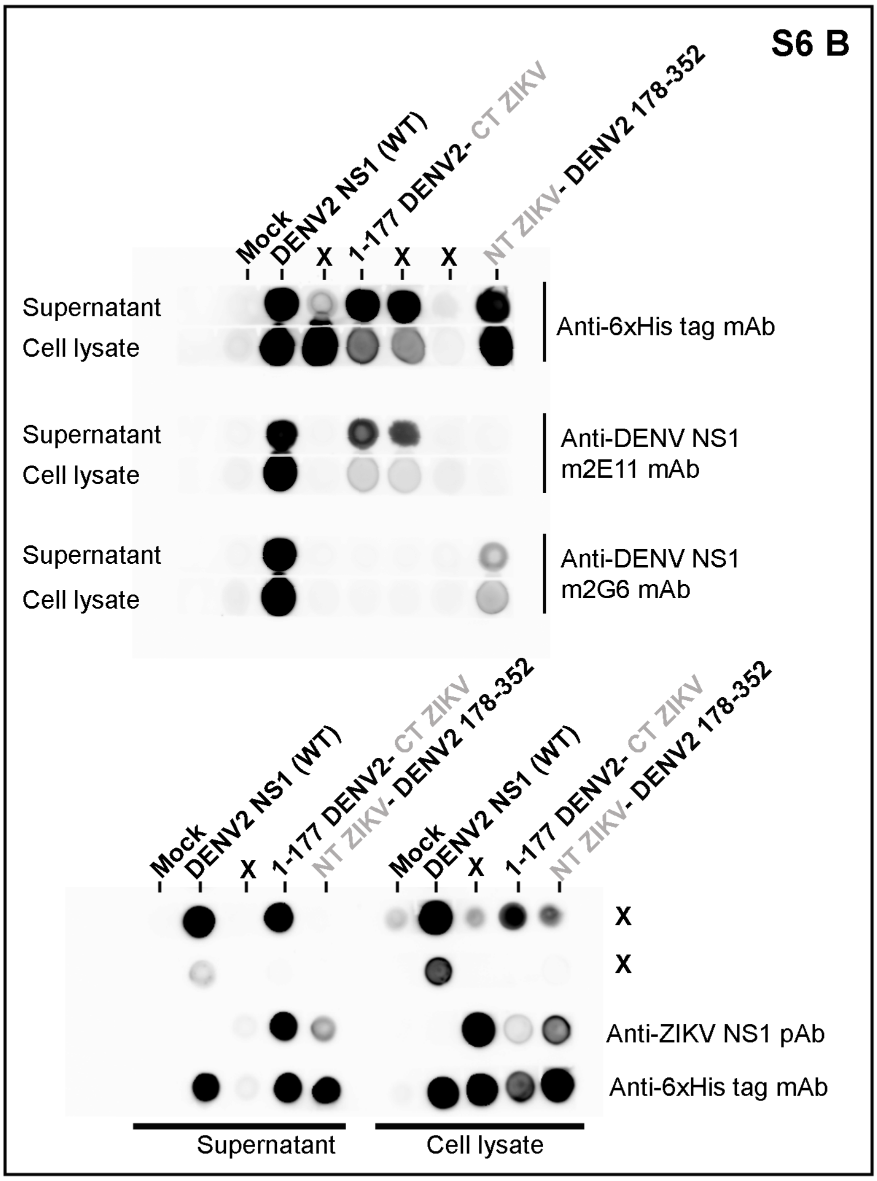

Original dot blot images to S6 Fig in the manuscript were shown in corresponding to each HEK293T-expressed recombinant wild-type(wt) DENV2,wt-ZIKV, and chimeric rDENV2-ZIKV NS1 with desired specific antibodies to confirm as indicated in S6 A and B.

Raw images for S6 Fig (cont).

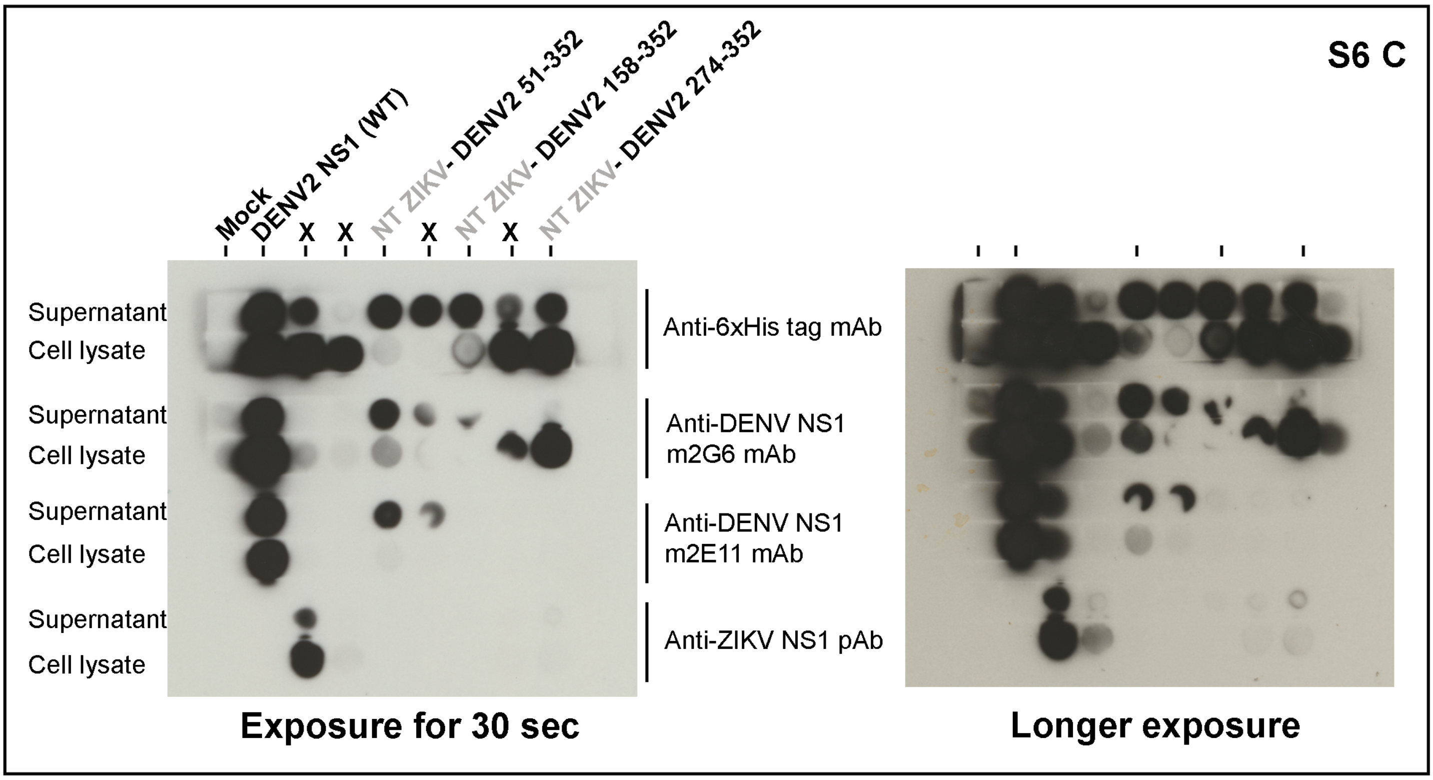

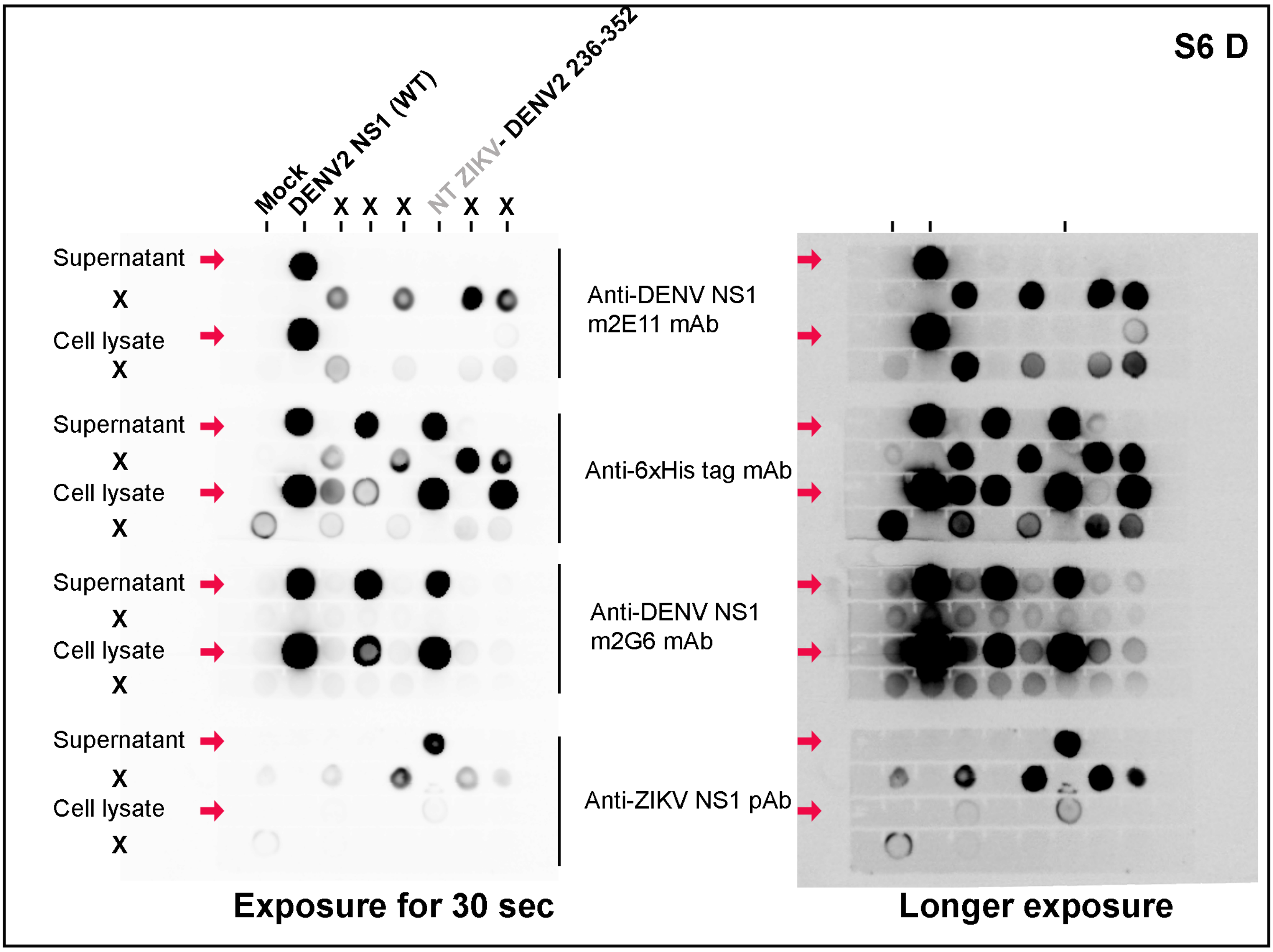

(Cont.) Original dot blot images to S6 Fig in the manuscript were shown in corresponding to each HEK293T-expressed recombinant wild-type(wt) DENV2,wt-ZIKV, and chimeric rDENV2-ZIKV NS1 with desired specific antibodies to confirm as indicated in S6 C and D.

Raw images for S7 Fig.

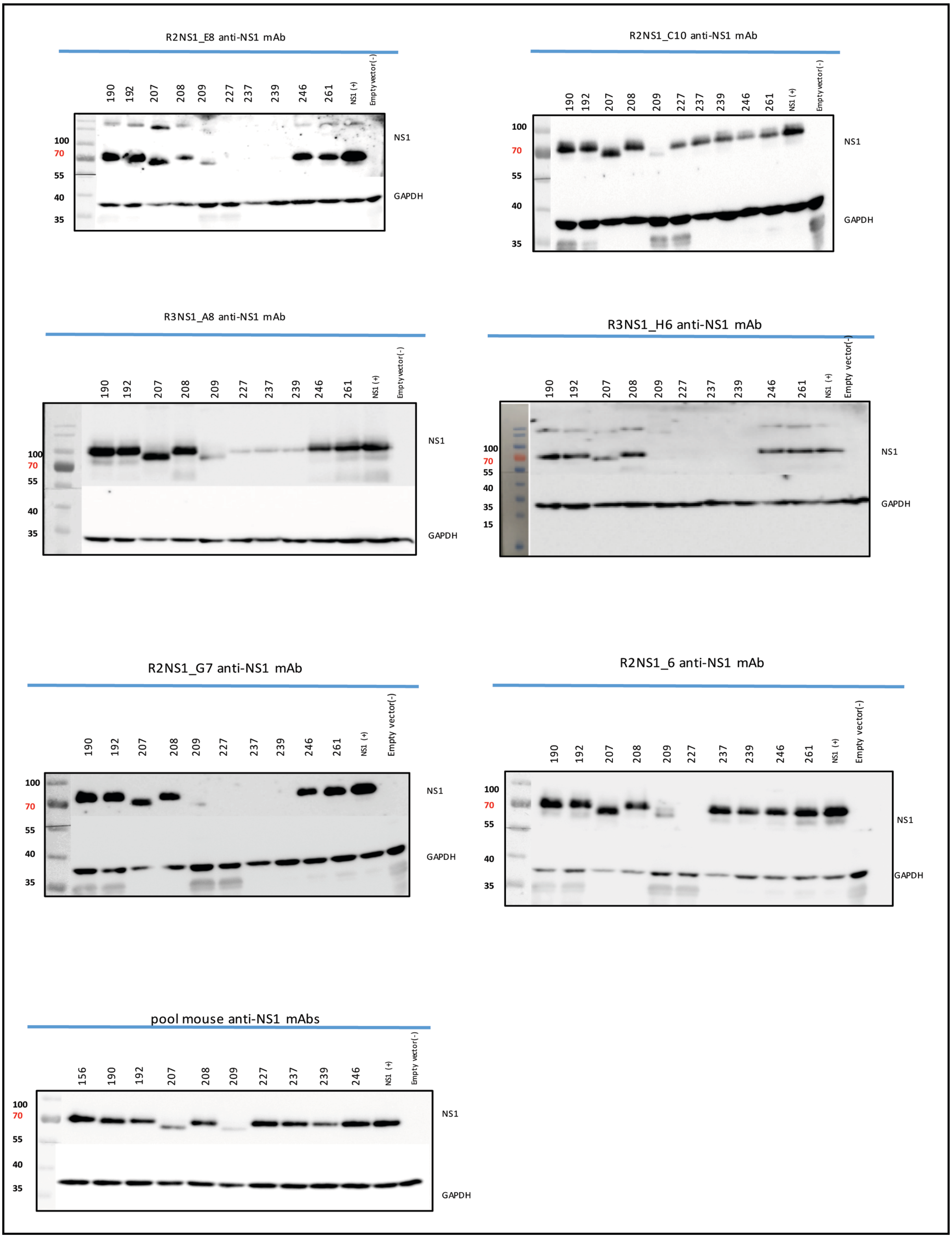

All alanine-substituted NS1 mutants at particular positions and tested mAbs are indicated above blots.

Raw images for S7 Fig. (Cont.)

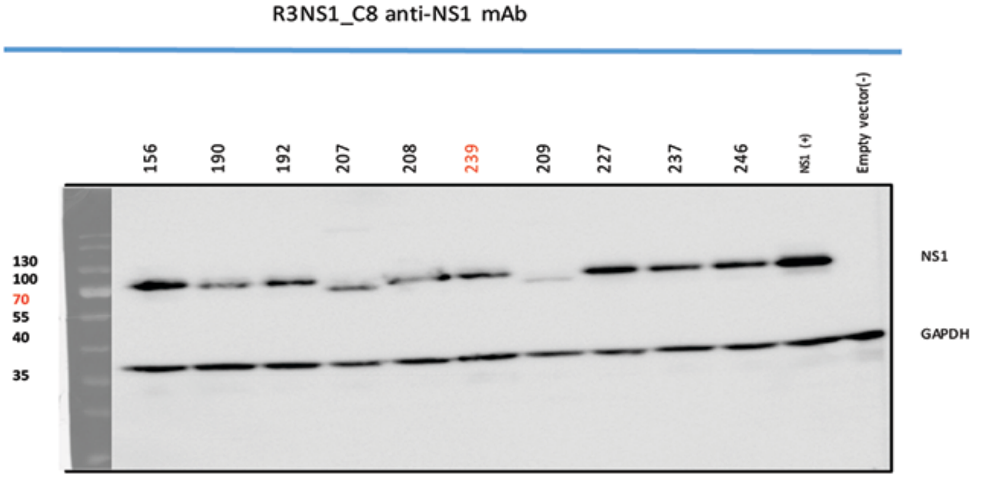

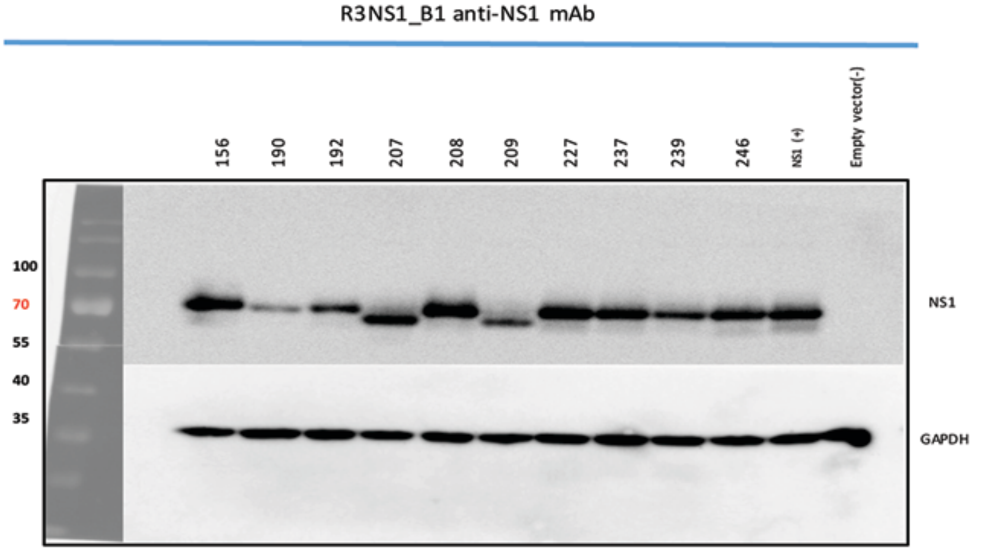

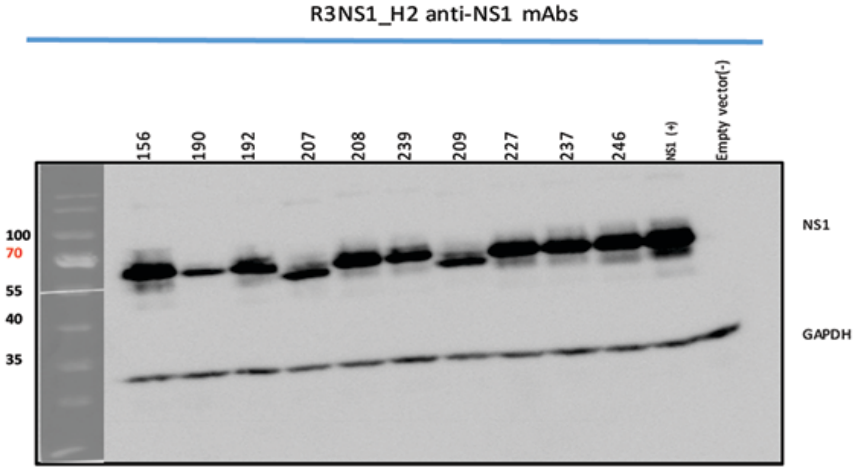

(Cont.) All alanine-substituted NS1 mutants at particular positions and tested mAbs are indicated above blots.
